# Keratin Associations with Synthetic, Biosynthetic and Natural Polymers: An Extensive Review

**DOI:** 10.3390/polym12010032

**Published:** 2019-12-23

**Authors:** Ricardo K. Donato, Alice Mija

**Affiliations:** 1 Institute of Macromolecular Chemistry, Czech Academy of Sciences, Heyrovského nám. 2, 162 06 Prague 6, Czech Republic; 2 Institute of Chemistry of Nice, UMR CNRS 7272, Université Côte d’Azur, University of Nice Sophia Antipolis, Parc Valrose, 06108 Nice CEDEX 2, France

**Keywords:** keratin, biomaterials, composite materials, polymer blends, functional proteins

## Abstract

Among the biopolymers from animal sources, keratin is one the most abundant, with a major contribution from side stream products from cattle, ovine and poultry industry, offering many opportunities to produce cost-effective and sustainable advanced materials. Although many reviews have discussed the application of keratin in polymer-based biomaterials, little attention has been paid to its potential in association with other polymer matrices. Thus, herein, we present an extensive literature review summarizing keratin’s compatibility with other synthetic, biosynthetic and natural polymers, and its effect on the materials’ final properties in a myriad of applications. First, we revise the historical context of keratin use, describe its structure, chemical toolset and methods of extraction, overview and differentiate keratins obtained from different sources, highlight the main areas where keratin associations have been applied, and describe the possibilities offered by its chemical toolset. Finally, we contextualize keratin’s potential for addressing current issues in materials sciences, focusing on the effect of keratin when associated to other polymers’ matrices from biomedical to engineering applications, and beyond.

## Contents

1.A brief historical context of knowledge and use of keratin based-materials2.Keratin’s structure and chemical toolset3.Sustainability and safety assessment4.Keratins extraction4.1.Oxidative and reductive extraction4.2.Steam explosion extraction4.3.Extraction with Ionic liquids and deep eutectic solvents5.Keratin sources and their distinctions6.Keratin-based biomaterials7.Keratin associations with other polymers7.1.Keratin associations with synthetic and biosynthetic thermoplastics7.1.1.Polyolefins7.1.2.Polyethylene glycol (PEG) and Polyethylene oxide (PEO)7.1.3.Poly(ethylene imide) (PEI)7.1.4.Polyacrylates, polyacrylonitrile (PAN) and polyacrylamide (PAM)7.1.5.Polyvinyl chloride (PVC)7.1.6.Polyvinyl alcohol (PVOH)7.1.7.Polyamide-6 (PA6)7.1.8.ε-Polycaprolactone (PCL)7.1.9.Polylactic acid (PLA)7.1.10.Polyhydroxyalkanoates (PHA)7.1.11.Thermoplastic polyurethanes (TPU) and polyurea-urethanes (TPUU)7.2.Keratin associations with elastomers and thermosets7.2.1.Butadiene copolymer rubbers7.2.2.Epoxy resins7.2.3.Urea-formaldehyde resins7.2.4.Phenol-formaldehyde resins7.3.Keratin associations with natural polymers and fibres7.3.1.Keratin associations with carbohydrates7.3.1.1.Cellulose7.3.1.2.Chitosan7.3.1.3.Alginate7.3.1.4.Starch7.3.2.Keratin association with other proteins7.3.2.1.Collagen and gelatine7.3.2.2.Soy and wheat protein7.3.2.3.Silk fibroin7.3.2.4.Associations between different keratin sources8.Conclusions and outlookAuthor ContributionsConflicts of InterestReferences

## 1. Brief Historical Context of Knowledge and Use of Keratin-Based Materials

**Figure 1:**
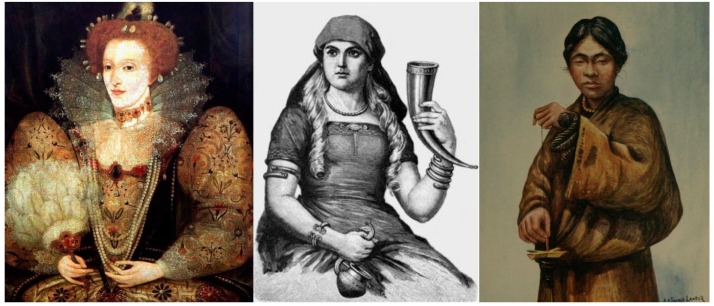
Keratin objects in history. Sixteenth century painting of Queen Elizabeth with a feather brisé fan (**left**); 19th century depiction of the Norse goddess Sif with a horn cup (**centre**); 1905 illustration of a Tibetan spinning wool by Landor A. H. S., from the collection “Tibet and Nepal” as digitised by the Internet Archive’s text collection (**right**).

Human civilizations have a long history of exploitation of keratin-rich tissues for the fabrication of daily life tools and ornaments, such as the use of horn sheaths as drinking vessels, mammalian and reptile skin as leather covers and clothing, feathers as different bedding materials and clothing, among many others [1,2] (Figure 1). From as early as the 16th century, there are reports of the use of pyrolyzed hair’s ash for blood clothing and wound healing in the “Compendium of Materia Medica” [3], and since the 19th century, there has been the knowledge that diverse dermic structures, such as hairs, feathers and hooves, consist of similar substances that were referred as “horn” or “keratin” [4].

The term “keratin” (from the Greek “kera” meaning horn) has been long used to refer to all proteins extracted from horns, claws and hooves, nails and other skin modifications. But further knowledge revealed that those were, in fact, associations of different keratins and other proteins. At this point, the term keratin was redefined as filament-forming proteins presenting specific physicochemical properties, which can be extracted from the cornified layer of the epidermis. The term was once again redefined more recently as all intermediate filament-forming proteins, with specific physicochemical properties produced in any vertebrate epithelia [4]. 

The research on keratins, keratin filaments and cornified structures began about 80 years ago. Only then was it recognized that these corneous tissues could vary from flexible to stiff due to small variations of these keratin molecules in the different cells, such as α- or β-structures, acidic or basic, with varied molecular weights (MWs) [5]. Later on, it was unveiled that the level of complexity of these variations among the keratin compositions, especially among different types of corneous tissues, was much more diverse than was prospected [6,7]. Cornified horse hairs were used to study the molecular structure of keratins by X-ray diffraction, presenting a quite regular structure that depends on the orientation of the X-ray. These studies revealed the α-helical structure or β-sheet structure of the keratin molecule’s rod domain, which is how the two main types of keratins are distinguished currently, i.e., α-keratins and β-keratins [4], and also the principal structural feature of all proteins [8]. Due to the further expanded knowledge about these molecules, their exploitation in areas such as the wool industry and for cosmetics and dermatology has only increased [9].

The understanding of the keratin structure and the comprehension that keratin extracts were in fact assembles of different keratin homologs (with different molecular weights) allowed the production of complex functional structures [10]. Moreover, between the decades of 1940 and 1970, after the publication of the first complete diagram of a hair fibre using X-ray diffraction and electron microscopy combined with oxidative and reductive chemical methods [11], a clearer understanding of the keratin chemistry led to the exponential growth of keratin materials’ and derivatives’ development [12], followed, in 1985, by the prospect of using keratin as the building block for new biomaterials’ development [13].

## 2. Keratin’s Structure and Chemical Toolset

A keratin protein is defined by a primary structure based on amino acid chains. These chains vary in number and sequence of amino acids, polarity, charge and size [14,15]. However, similarities exist in their structure independent of the species of animal or function [16]. Small modifications in the keratin’s amino acid sequence cause significant properties’ modification, since these sequences determine the whole molecular structure and the nature of the bonds (e.g., covalent or ionic) [17,18].

Keratins were classified into two distinct groups considering their structure, function and regulation: i) “Hard” keratins forming ordered filaments embedded in a cysteine-rich proteins’ matrix, presenting a compact and hard structure; ii) “Soft” keratins forming loosely-packed bundles of filaments and with the function to grant elongation and stress release [19]. The structural subunits of both epithelial and hair keratins, which differ in molecular weight and composition, were designated as types I (acidic) and II (neutral-basic), forming heterodimers that further polymerize into 10 nm intermediate filaments [7,20]. 

**Figure 2:**
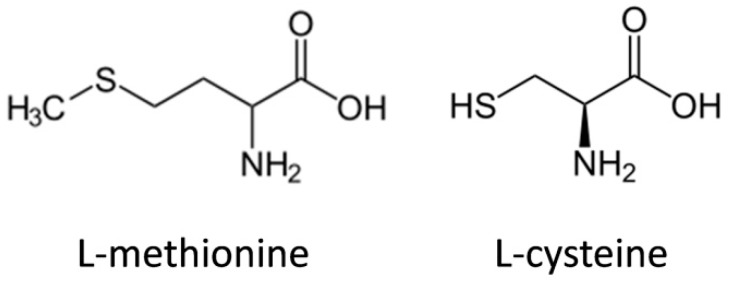
Sulphur-containing amino acids in keratin.

Within this context, the sulphur-containing amino acids, methionine and cysteine (Figure 2), present even greater influence due to their role in establishing intra- or intermolecular disulfide bonds. These disulfide bonds are formed by connecting two sulfhydryl functionalities of two amino acids (such as two cysteines) enzymatically via the enzyme sulfhydryl oxidase [21]. The role of disulphide bonds is so important in keratin’s structuration, due to the necessity of structural integrity, that the adaptive evolution processes led to the convergent evolution of cysteine-rich proteins in animals’ hair and feather [22].

The mechanical properties of keratin-based materials were not comprehended for a long time; however, their bond lability has been revealed and many of the reinforcing mechanisms understood. Keratin polymerizes into intermediate filaments consisting of a central elongated α-helical domain, flanked by a globular head (*N*-terminal), and a tail (C-terminal) domain [23]. The extensibilities of different types of intermediate filaments (including keratin) were determined by cleaving these filaments laterally with an AFM tip, finding a maximum breaking strain of 260% [24]. This large extensibility was proposed to be made possible by a transition of the central α-helical coiled coil rod to an elongated β-strand structure [25], which was further demonstrated for keratin and hair fibres (matrix embedded keratin) under mechanical stress [26]. Hard α-keratin is a tough composite material that forms structures such as wool, hair, hooves and claws in mammals. This composite consists of keratin microfibrils, (very similar in structure to the intermediate filament), embedded in a sulphur matrix. The breaking strain of hard, wet α-keratin fibres, such as hair and wool, is about 45% and their Young’s modulus is about 2000 MPa. Moreover, *α* to *β*-conversion has also been demonstrated to be reversible in hydrated, hard keratin, such as wool [27].

On the other hand, the amino acid chains of β-keratins, which are characteristic of hard-keratinized and hard-cornified modified epidermis in reptiles and birds, are shorter than those of α-keratins [4]. For example, in the β-keratin of the emu feather, only 32 amino acids form the central rod domain, 23 amino acids form the head domain and 47 amino acids form the tail domain [28], while α-keratins can present hundreds of amino acid residues [29]. 

**Figure 3:**
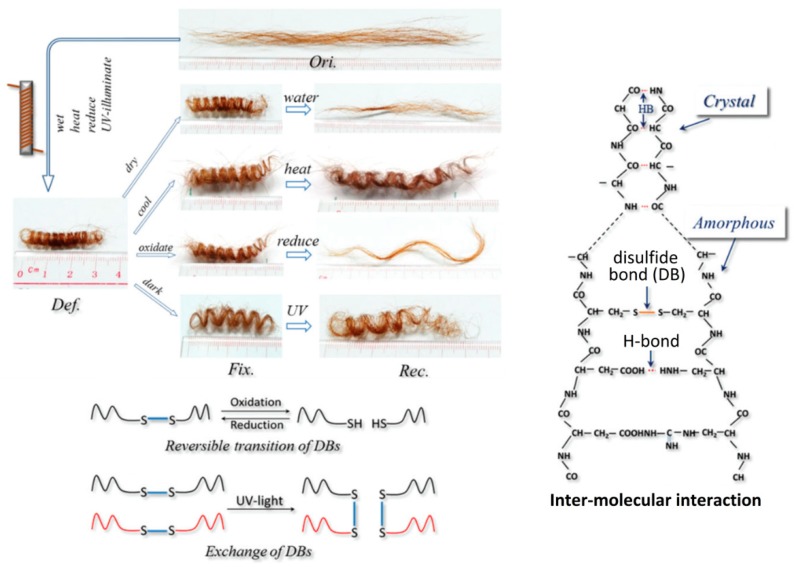
Camel hairs under four different stimuli: Original (Ori.), deformed (Def.), fixed (Fix.), recovered (Rec.), induced by water, heat (85 °C), redox (NaHSO_3_/H_2_O_2_ solutions) and UV-light (254 nm) (**top-left**). Hierarchical structure, inter-molecule bonds and crystals formed within the hair keratin (**right**). Schematic representation of the oxidation/reduction effect forming reversible disulfide bonds (DBs) and exchange of DBs among macromolecules under UV-light. Adapted from [30] with permission from The Royal Society of Chemistry.

This broad chemical tool-set and structural variation allows the application of keratin with quite varied functions. For example, the flexible but resistant hair α-keratin allows for very effective multi-responsive smart materials, since it presents isolated shape-memory responses to oxidation/reduction, moisture, temperature and light [30] (Figure 3), while the stiff and densely packed avian β-keratin can present tensile moduli and tensile strengths of approximately 3.6 GPa and 203 MPa, respectively, acting as an effective filler for polymer composites [31].

## 3. Sustainability and Safety Assessment 

The dramatic increase in polymer production and consumption—348 million metric tons worldwide in 2017—came together with major environmental challenges, especially for areas such as packaging, since about 40% of all thermoplastics were produced in Europe. About 40% of worldwide plastic production is used in one-way products and 32% of those leak into the environment; thus, they have become a major environment contaminant [32,33].

**Figure 4:**
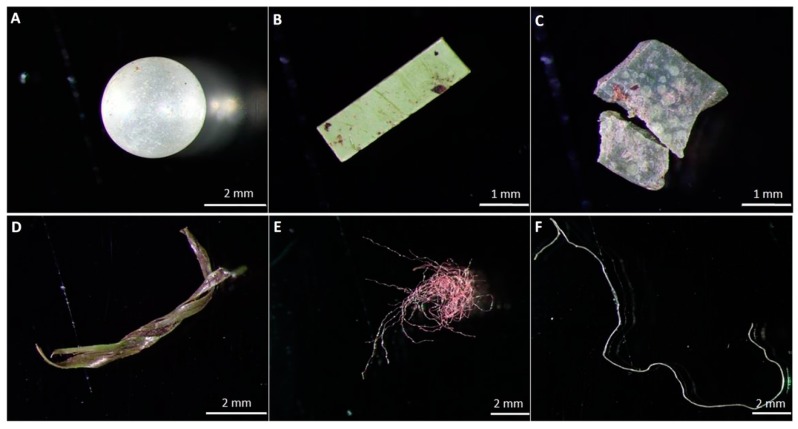
Examples of microplastic polymer particles (MPPs) of various morphologies; (**A**) polyethylene sphere, (**B**) polyvinylchloride fragment, (**C**,**D**) polyethylene fragments, (**E**) polyester fibre, (**F**) polypropylene fibre. “Reprinted from ref. [36]. © The Authors, some rights reserved; exclusive licensee American Association for the Advancement of Science. Distributed under a Creative Commons Attribution Non Commercial License 4.0 (CC BY-NC) http://creativecommons.org/licenses/by-nc/4.0/”.

In addition to these almost 13 million metric tons of direct plastic contaminants that enter the environment each year [34], the usually durable commodity polymers also undergo incomplete disintegration caused by ultraviolet (UV) radiation, mechanical abrasion, and biological degradation [35]. This process produces microplastic polymer particles (MPPs), which are <5 mm fragments (fibres and spheres) resulting from their incomplete degradation (Figure 4), causing direct harm by their bioaccumulation and also indirect harm due to the toxic additives and microorganisms they carry on their large surface areas, which enter the food web and consequently, human food [36,37].

Bio-based polymers such as keratin are also hard-degrading fibrous proteins, insoluble in water and organic solvents, and may cause environmental problems, especially because important quantities of this by-product are mass-produced by the poultry industry [38]. However, contrarily to the fossil-based synthetic polymers, there are plenty of keratin-degrading microorganisms, such as bacteria, archaea, actinomycetes and fungi, that employ keratinases to attack keratin, allowing composting processes [39]. Moreover, their degradation products are majorly peptides and amino acids that return to the biocycle and act as biofertilizers, which, most importantly, also avoids the formation of microplastics [40,41]. 

Considering the latter, it seems inconceivable that the waste of many keratin-rich industrial side-streams, such as poultry feather with about 90% keratin content, has been massively produced and only landfilled or incinerated in industrialized countries such as the USA, Australia and Japan, but also improperly dumped in road side disposals in developing countries such as India, causing major environmental and health issues [38,42]. Although, when properly managed, poultry feather waste can be used as nutrient source for soil recycling [43], prior to composting, it can also offer plenty of opportunities as a source of functional biomaterials, applicable in many different areas [44], which is especially appealing for the one offering the largest environmental threat, the packaging industry [45].

## 4. Keratins Extraction

In order to better understand the structure of keratins and their potential applications, the proteins of cornified organs can be extracted through the use of various solvents and denaturing agents. However, keratin does not behave like other proteins, and usual methods for dissolving proteins are generally ineffective for solubilizing it. Nevertheless, under controlled conditions, especially under low pH and in the presence of reducing/oxidizing agents, it becomes more water-soluble and chemically reactive due to its disulfide (–S–S), amino (–NH2) and carboxylic acid (–COOH) moieties [46].

The most common methods of keratin extraction are discussed below; moreover, more extensive descriptions and comparisons among the methods may be found elsewhere [47]. 

### 4.1. Oxidative and Reductive Extraction

One of the first studies to solve this issue of insolubility was a patent issued by John Hoffmeier in 1905, which described a process for extracting keratins from animal horns using lime [48], followed by many other methods using oxidative and reductive chemistries [20]. Similar approaches are still in use today, e.g., the use of oxidative agents such as peracetic acid [49]; thermo-chemical treatments with various reducing agents, such as 2-mercaptoethanol, dithiothreitol, sodium m-bisulphite and sodium bisulphite followed by NaOH treatment [50]; solubilisation with potassium cyanide, thioglycolic acid and sodium sulphide followed by precipitation with ammonium sulfate [51]; among many other variations of the so-called Shindai method [52], as further discussed elsewhere [44].

### 4.2. Steam Explosion Extraction

With the intention to avoid the initial chemical treatment, wool fibres were treated by steam explosion, which is a physical treatment involving an instant discharge of high-pressure steam in a sealed container. The application of this method helped considerably to break the keratin disulfide cross-links and showed no evident changes in the fibre chemical composition, however, by increasing the steam pressure, a decrease in the fibre crystallinity, thermal decomposition energy, and changes in the sulphur-containing groups were observed in the post-treated wool [53].

**Figure 5:**
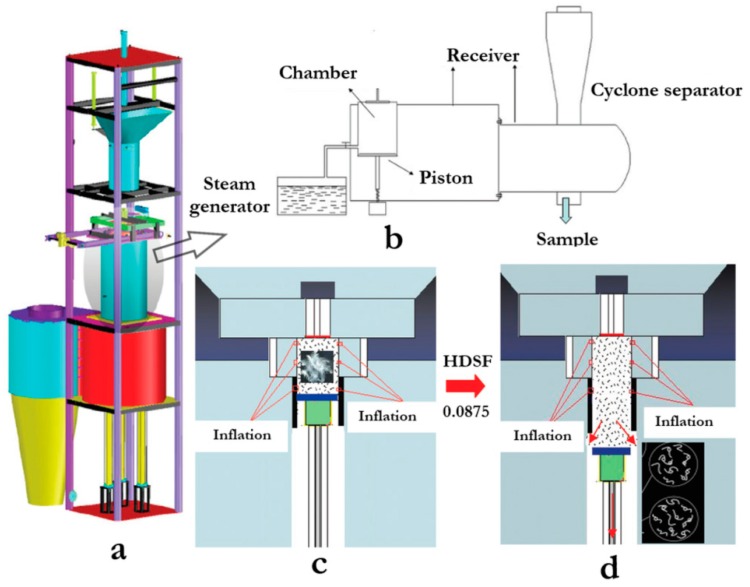
Schematic representation of high-density steam flash-explosion, displaying the structure for catapult explosion mode, composed of a cylinder and a pistol (**a**,**b**). The process presents two phases: (**c**) steam pressurization, where the two parts are tightly coupled, and (**d**) explosion, where the piston is driven by three pneumatic linear actuators and the kinetic energy of the steam and material, bursting out of the cylinder. Adapted from [54] with permission from The Royal Society of Chemistry.

Later on, this method was optimized by exposing the extraction source to high-temperature steam and forcing the steam into the material’s composition, followed by explosive decompression (completed in milliseconds), denominated as high-density steam flash-explosion [54,55] (Figure 5). 

Yang et al. [56] used this process followed by alkali treatment for the extraction of keratin from duck feathers. They optimized the conditions as 1.6 MPa steam explosion for 1 min, followed by extraction with 0.4% NaOH (NaOH_sol._/feather ratio = 20/1 (*v*/*w*)) at 25 °C for 1 h. Under these conditions, the extraction rate of feathers was 65.78% and the yield of keratin was 42.78%. The process was very effective to disrupt the disulfide and hydrogen bonds, even with large amounts of feathers (~100 g), however, it also resulted in a relatively low keratin yield, caused by macromolecular chains fragmentation and the loss of the ordered structure [56].

### 4.3. Extraction with Ionic Liquids and Deep Eutectic Solvents

Alternative approaches to better conserve protein integrity after extraction have been attempted, among those, the use of Ionic liquids (ILs) and deep eutectic solvents (DES) has been extensively explored. The ILs are organic salts with a melting temperature (*T*_m_) ≤ 100 °C, presenting ionocovalent structures, constituted of pairs of counter ions (forming physical macrostructures dependent on the concentration) and are often liquid at room temperature [57]. DES are low transition temperature mixtures consisting of at least one H-bond donor and one H-bond acceptor counterpart, usually consisting in an organic salt together with a H-bond donor. Both ILs and DES often present extremely low volatilities, and their properties can be adjusted by selecting the nature and ratio of the ion and the hydrogen bonding pairs [58]. They have been used as a mild option for chemical treatments to extract keratin and other natural polymers from their raw sources, especially due to their capacity to keep (or tune) the properties of the original polymer and also due to their recyclability [59].

Li et al. [60] used this approach to regenerate wool keratin to form films, which were prepared from the wool keratin/IL solutions through the addition of water, methanol or ethanol as coagulation solvents. They demonstrated that an IL presenting an unsaturated cation side chain (1-allyl-3-methylimidazolium chloride) had a higher solubility for wool keratin fibres than that of a similar IL with saturated alkyl cation side chain (1-butyl-3-methylimidazolium chloride). Interestingly, XRD data also confirmed that the regenerated films exhibited a β-sheet structure and the disappearance of the α-helix structure. 

**Figure 6:**
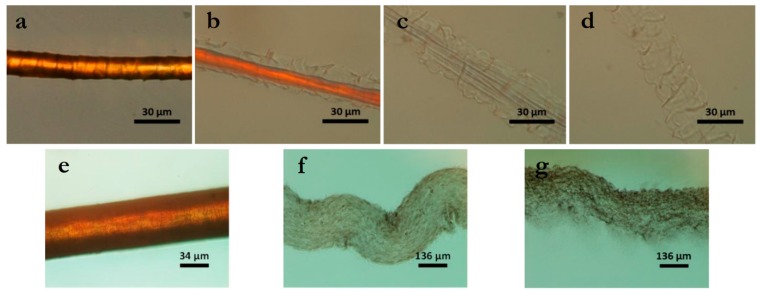
Polarized optical microscope (POM) images of a wool fibre in [Bmim]OAc at 120 °C: (**a**) original wool fibre; for (**b**) 5 s; (**c**) 50 s; (**d**) 100 s; and (**e**) 180 s. POM images of a black hair fibre in [Bmim]OAc at 120 °C: (**a**) original fibre; for (**b**) 25 min; and (**c**) 90 min. Adapted from [61].

Trying to better understand this dissolution mechanism, Byrne et al. [61] performed an in situ dissolution at 120 °C of single wool and hair fibre in 1-butyl-3-methylimidazolium acetate IL and observed it using polarized optical microscopy. They noticed that initially, the cuticle swells (outer layer of the fibre) followed by swelling of the cortex (inner layer of the fibre, Figure 6a–d). During this process, the crystallinity in the cortex was destroyed (becoming transparent), suggesting that dissolution starts from the cortex. Within 3 min, the cortex was completely dissolved, leaving behind the cuticle that took about 1 h to fully dissolve. The authors suggest that the long time required to dissolve the cuticle is connected to the higher disulphide bond content present in this region of the fibre. They applied the same experiment for a darkly pigmented hair, since in this case, the pigmented fragments could be observed, better revealing both inner and outer fibre parts. Similarly to the wool, the hair suffered a considerable swelling process, also showing that the dissolved cortex remains inside the swelled cuticle until it is also dissolved (Figure 6e–g).

For chicken feathers extraction, a more hydrophobic IL was used, 1-hydroxyethyl-3-methylimidazolium bis(trifluoromethanesulfonyl)amide, and the extracted keratin was soluble in water, allowing an easy isolation of the product and recycling of the extraction system [62]. Chen et al. [63] applied 1-butyl-3-methylimidazolium chloride together with Na_2_SO_3_ to cleave the chicken feathers disulfide bonds and dissolve the keratin. They were able to reach about 97 wt.-% keratin extraction with final keratin regeneration of 75 wt.-%. Both the IL and Na_2_SO_3_ could be recycled in the process. Also using 1-butyl-3-methylimidazolium chloride, Liu et al. [64] demonstrated the preparation of chicken feather-based particles. The particles based on the regenerated keratin presented reduced crystallinity (considerably lower β-sheet formation) and were much more hydrophilic (water contact angle reduction from 138° to 76°) in comparison to the pristine feather.

Wang et al. [65] used imidazolium ILs with phosphonium anions to dissolve wool keratin (at 130 °C for 1.5 h). The authors demonstrated that although the dimethylphosphonium anion presents a slightly weaker dissolving strength than the acetate anion, the first one has the ability to better conserve the crystallinity of the native keratin, especially the α-helix, which was apparently fully conserved while the β-sheets were partially unpacked.

Although most ILs discussed present imidazolium-based cations, McFarlane et al. [66] demonstrated that ammonium- and choline-based ILs can also dissolve up to 45wt.-% of turkey feather keratin (at 130 °C for 10 h, without the addition of any other chemicals), and from this dissolved amount, up to 51 wt.-% could be regenerated by water extraction and precipitation, without causing significant chemical changes. Moreover, Zhang et al. [67] used ILs with diazabicyclo-based cations to dissolve goat wool (at 120 °C for 3 h). The relative crystallinity content of α-helix and the amount of disulphide-bond remaining after dissolution varied considerably and were completely dependent on the structures of both the IL’s cation and anion.

**Figure 7:**
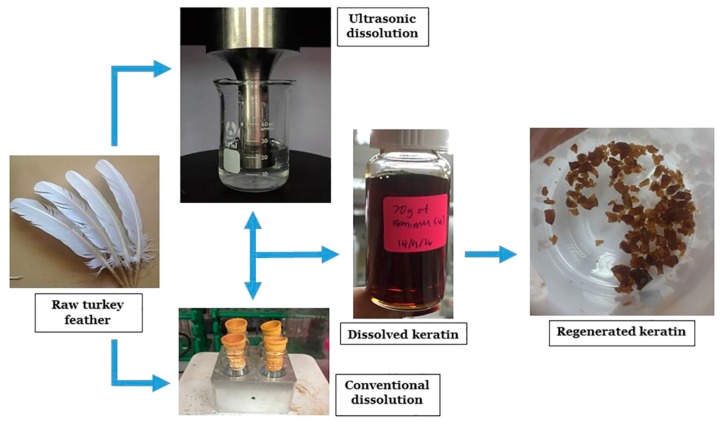
Representation of the dissolution and regeneration processes of raw turkey feather through ultrasonic-assisted and conventional methods. Reprinted from [68], with permission from Elsevier.

Moreover, Yusof et al. [68] studied the optimization of these keratin extraction processes with IL by comparing the use of conventional mixing processes and the application of ultrasonic techniques for the dissolution of turkey feather in ILs or ILs associated with co-solvents. They demonstrated that the application of low-frequency high-power ultrasonic irradiation significantly improves the dissolution rate of feather keratin, decreasing the dissolution process time from 2 h to about 20 min, both applying pure 1-butyl-3-methylimidazolium chloride IL or a 2.0 M solution of IL in dimethyl sulfoxide. No major chemical damage of the polypeptide chains was observed with the applied ultrasonic method, with the keratin presenting only minor structural changes after the extraction process (Figure 7).

Concerning the use of DES, Yuan et al. [69] were able to dissolve wool fibres in choline chloride-urea (1:2) DES, 35.1 mg/g at 130 °C in 5 h, to produce regenerated wool keratin. Similarly to the effect of ILs, they observed that DES mainly dissolved the wool cortex layer. The process produced a considerable decrease in the amount of α-helix, while the content of β-sheet and disordered structures increased, indicating α-β transition and some chain fragmentation. Boulos et al. [70] applied a similar approach, but using a choline chloride-urea DES with a 2:1 molar ratio. They also dissolved 5 wt.-% of wool in DES, although in a harsher and shorter process (170 °C for 30 min); however, the authors did not present data concerning the process influence in the keratin crystallinity and general morphology. 

Wool (commercial, without described source) has been also successfully dissolved in choline chloride-oxalic acid (1:2) DES, 5 wt. % at 125 °C for 2 h. Tang et al. [71] observed that the dissolution process provoked the wool’s disulfide bonds cleavage and disruption of the α-helix structure, producing a regenerated keratin with molecular weight between 3.3 kDa and 7.8 kDa. The same group also dissolved rabbit hair in choline chloride-oxalic acid (1:2) DES, 1 wt. % at 120 °C for 2 h, reaching 88 wt.-% solubility of the initial mass. The keratin produced presented a molecular weight ranging from 3.8 to 5.8 kDa and with clear disulfide bonds cleavage and α-helix structure disruption [72].

Recently, feathers were processed with an aqueous DES to produce a uniform keratin feedstock. The authors propose a DES composed of non-toxic sodium acetate and urea, and a small amount of water. The processing conditions were optimized in terms of keratin yield of regenerated keratin (E.-M. Nuutinen, P. Willberg-Keyriläinen, T. Virtanen, A. Mija, L. Kuutti, R. Lantto, A.-S. Jääskeläinen, RSC Adv., 2019, **9**, 19720-19728).

## 5. Keratin Sources and Their Distinctions

Since keratin is a tough, fibrous and insoluble material that protects animals’ organs and prevents the loss of bodily fluids, it is consequently also one of the most abundant biopolymers available. Keratin sources are vast, ranging from hair, wool, horns, nails, claws, and hooves of mammals (α-keratins) to bird feathers, beaks and claws (β-keratin), as only a few examples [73,74]. However, three sources, namely wool, hair α-keratins, and feather β-keratin, have been overwhelmingly more explored and described in the literature due to their vast availability as side streams of slaughterhouse, tanning, fur processing and poultry industries, and their consequent potential for large-scale exploitation [75].

**Figure 8:**
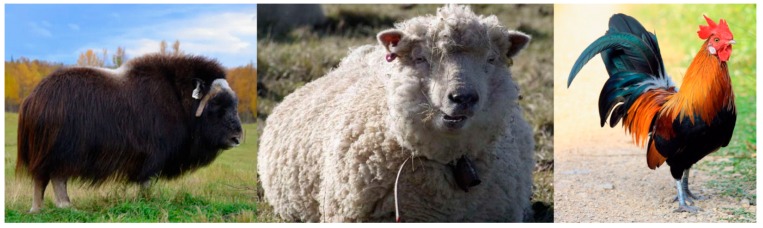
Photographic images of a “hairy” Alaskan musk ox (**left**), a “woolly” merino sheep (**centre**) and a “feathery” rooster (**right**).

Wool and hair are unique traits of mammals, while feathers are only found in avian species (Figure 8), with the exception of long-extinct dinosaur species [76]; consequently, the keratins obtained from these different sources also present significant variations in amino acid composition, molecular weight, and protein secondary structures. While wool and hair keratins present polydisperse proteins with molecular weights between 10 and 75 kDa mainly constituted of α-helix structures, feather keratins consist of low polydisperse proteins with a molecular weight of ~10 kDa mostly structured in β-sheets [4,77]. These structural differences are due to the different biosynthesis pathways of α- and β-keratins [78], which are also most likely due to their difference in function.

**Figure 9:**
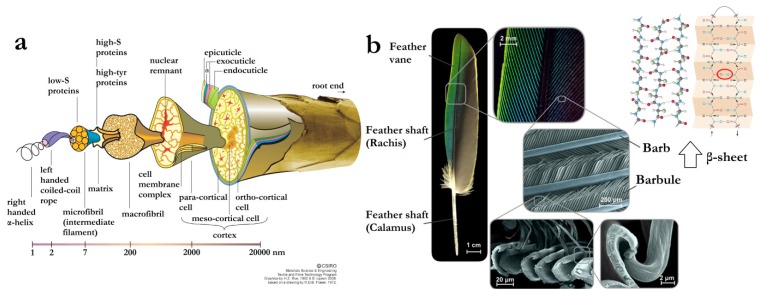
(**a**) Schematic diagram depicting the internal structure of a wool fibre and the scale of its components, adapted with permission from CSIRO [81]; (**b**) photographic images and micrographs showing a bird’s feather structure and a keratin β-sheet as the major composition component of the feather, adapted from [78,80], with permission from Elsevier.

Animal hair and wool present excellent elasticity and thermal insulation, properties that are ascribed to their hierarchical structure, with macro and micro-fibrils and helical coils, which are wrapped in the outside cortex and cuticles [30]. More specifically, wool is structured in three main components: a hydrophobic exterior lipid layer, found on the cysteine rich epicuticle and covalently bound via thioester moieties; the outer layer cuticle cells (approximately 0.5-mm thick), constituted of the epicuticle, exocuticle and endocuticle, differing from human hair (comprising of up to 10 cuticle layers); and the central core (composed of a medulla surrounded by a cortex), consisting of a large number of cortical cells (with high-sulphur macro- and low-sulphur micro-fibrils) (Figure 9a) [70].

On the other hand, avian feathers are designed for maximum performance with a minimum-weight penalty, being structures in ingenious combinations of components to optimize major flight requirements, such as lift, stiffness, aerodynamics and damage resistance. This is achieved by their being majorly composed of β-keratin and possessing a particular design divided into two main parts, a central shaft (rachis and calamus) for stiffness and lateral vanes (barbs and barbules) for capturing air. A flat surface is formed by branching between barbs from the shaft and barbules from the barb, held together by microhooks at the end of the barbules (Figure 9b) [79]. Details about these structures and the reflex in the success of birds’ flying ability were brilliantly described by Meyers et al. [80].

**Figure 10:**
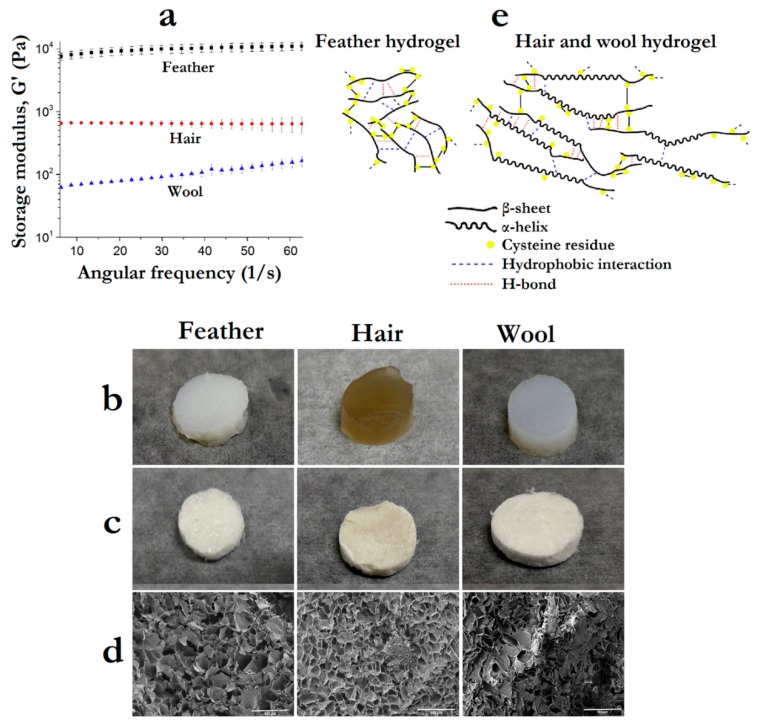
Dynamical mechanical and structural differences between networks produced with different keratin sources, namely feather, hair and wool: Rheological data of hydrogels showing the storage modulus (G’) in function of the shear frequency applied (**a**); photographic images of hydrogels (**b**); photographic (**c**) and SEM images (**d**) of scaffolds obtained by freeze-drying the hydrogels; proposed self-assembly of keratin hydrogels indicating the disulfide bonds, hydrophobic interactions and hydrogen bonds within the keratin networks (**e**). Adapted from [46], with permission from Elsevier.

As in nature, these functional differences between keratin sources need to be taken into account for designing keratin-containing systems. This was clearly demonstrated by Wu et al., [46] by using three different keratin sources (merino wool, human hair and chicken feather) to produce hydrogels and scaffolds, comparing their rheological, physical and biocompatibility properties. They observed that hydrogels prepared with chicken feather keratin display considerably higher storage modulus (7.6–11 kPa) in comparison to those prepared with hair (~0.7 kPa) or wool keratin (0.06–0.16 kPa) (Figure 10a). On the other hand, feather keratin hydrogels presented a much worse swelling capacity (1500%) than hair or wool keratin hydrogels (>3000%) (Figure 10b), affecting also the structure of scaffolds formed by freeze-drying the hydrogels (Figure 10c,d).

The authors attribute the results to the lower molecular weight and β-sheet conformation of feather keratin that could facilitate the self-assembly of rigid hydrogels through disulfide bond re-oxidation, while the higher molecular weight and α-helix conformation in hair and wool keratins led to more flexible/weaker hydrogels (Figure 10e). The cell proliferation on the formed scaffolds, using fibroblasts, was affected by the use of different keratin sources, where the highest proliferation rate was observed for chicken feather keratin-based scaffolds. Thus, in this case, feather keratin was the most suitable source to produce mechanically robust biomaterials that can promote cell proliferation for wound-healing biomaterials [46].

## 6. Keratin-Based Biomaterials

The use of keratin to produce functional biomaterials is widespread in different areas, ranging from applications in biomedicine [20,82] and drug-delivery [83], as natural polymer flocculants [84] and absorbents [85], in biolubricant formulations [86], to bioelectronics [87].

**Figure 11:**
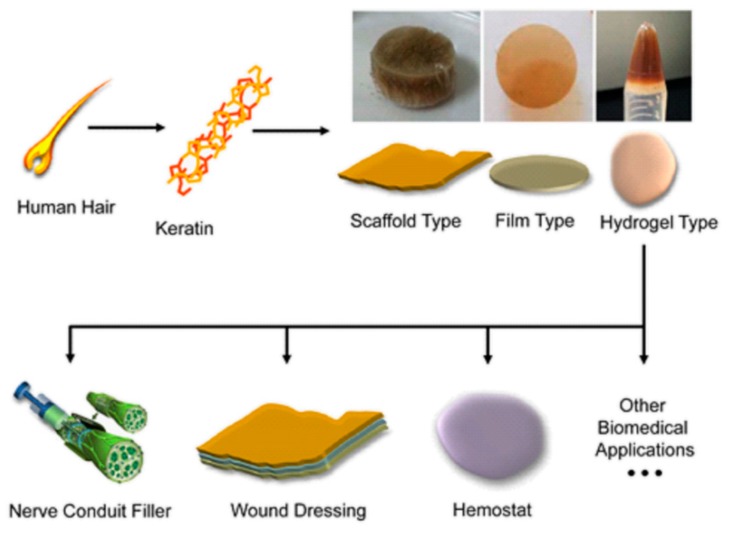
Human hair keratin applications as a medical biomaterial. Reprinted from [90], with permission from Springer.

Extracted keratin proteins have been especially explored in the preparation of materials for medical applications, due to their intrinsic ability to self-assemble and polymerize into porous fibrous scaffolds, producing reproducible architecture, dimensionality and porosity that are crucial for controlled cell growth [88]. Moreover, the keratin structure is also tuneable on a macroscale, since keratin solutions can be transformed by electrospinning into three-dimensional fibrous scaffolds [89]. Consequently, reports of the successful use of keratin can be found in a variety of biomedical applications, e.g., as nerve conduit filler for peripheral nerve regeneration, hydrogels or films for wound healing, hemostatic agents, and scaffolds for tissue regeneration (Figure 11) [90].

However, concerning thermomechanical performances and cost-effective processing, although presenting considerable sources for obtaining prime material, the largest hurdles preventing keratin-based biomaterials from replacing “commodity” fossil-based polymers, e.g., PP and PE, are their poor processing and post-processed mechanical characteristics [20]. The melt processing of neat keratin requires the use of redox agents and large amounts of plasticizers [91], which affect the material’s final mechanical properties. Furthermore, the additive-less production of neat keratin bulk materials has been described, however, it demands high pressures and temperatures [92], which also limits their production. In this matter, polymer blending is one of the most feasible options, since it allows the preservation of the excellent biological activity of keratin and the addition of the mechanical characteristics of other natural polymers [93,94] or other synthetic polymers with well-established processes [95,96], since the complementary polymer can substitute the functions of the plasticizers during processing and act as filler, coupling agent or crosslinker reinforcing the final blend/composite material. 

## 7. Keratin Associations with Other Polymers

**Figure 12:**
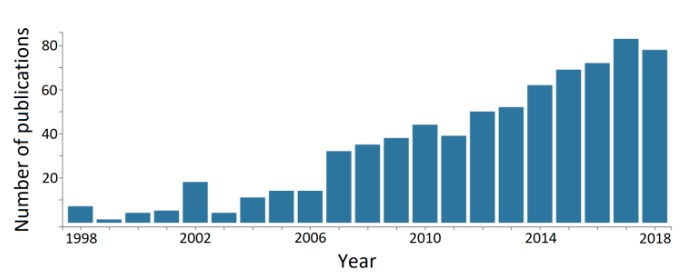
Number of publications in the last two decades concerning keratin associations in materials. The data were retrieved from Web of Science (WOS) on 1 August 2019 using the following criteria: Topic (keratin association) or (keratin blend) or (keratin composite) or (keratin polymer). The results were refined by the WOS categories associated to nanotechnology, materials, polymer, chemical and physical sciences.

The idea of using available keratin sources, such as feather and wool, as fillers for (or associated with) other polymer networks, is far from new. One clear example is a conference held in 1955 by the U.S. Army Quartermaster Corps about the utilization of chicken feathers as filling materials, with the contributions further compiled and published as a book by the U.S. National Academies of Sciences, Engineering and Medicine [97]. However, those ideas seem to have followed the wave of economic growth and awareness of the effects of unsustainable growth in the early post-World War II era [98], receiving decreased attention in the following decades, but experiencing a resurgence during the last two decades or so (Figure 12).

### 7.1. Keratin Associations with Synthetic and Biosynthetic Thermoplastics

Thermoplastic polymer associations may lead to blend formation (physical blending) or copolymer formation (chemical blending), which has generally been the most affordable approach to correct or add polymer properties to a polymeric system. However, polymers are usually immiscible, forming incompatible blends, and their miscibility is directly dependent on the polymers’ functional groups or the addition of proper coupling agents. Thus, generally, polymer blends present very specific properties related to the polymer pairs, which also allows a broad range of property outcomes [99]. 

**Figure 13:**
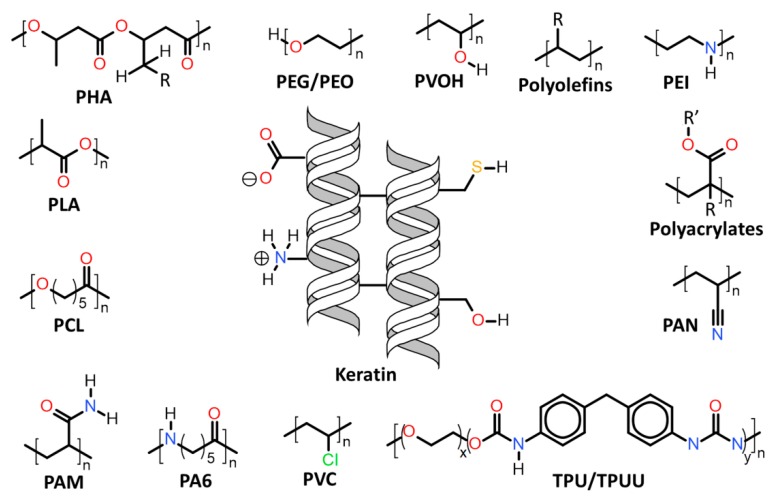
Schematic representation of the synthetic and biosynthetic polymers discussed, evidencing their available functionalities for interacting with keratin.

Herein will be discussed the associations of keratin with synthetic and biosynthetic thermoplastic polymers, categorizing the available literature by polymers/polymer class used in association with keratin. The polymers’ generalized structures and different functionalities available are represented in Figure 13, and at the end of this section, Table 1 summarizes the main processes used and the thermomechanical properties achieved with the keratin/thermoplastic polymer blends.

#### 7.1.1. Polyolefins

Polyolefins are polymers produced by alkene (olefin) polymerization, i.e., an unsaturated chemical molecule presenting at least one carbon-carbon double bond, which includes the vastly produced polyethylene (PE), polypropylene (PP) and polystyrene (PS) [100]. Considering the more than 300 commercially available polyolefins, they account for more than 50 wt. % worldwide-produced polymers, with about 178 million tons produced in 2015 alone [101]. The packaging industry is one of their main consumers, due to their good mechanical properties, thermal stability, good barriers properties to carbon dioxide, oxygen and aromatic compounds, and also their large availability and relatively low cost. Consequently, they are also among the main contributors for the huge environmental impact caused by, in particular, the packaging industry; and the blending of polyolefins with available natural polymers has been a feasible option to tackle this issue [102]. Very recently, Shavandi and Ali published a review summarizing the processing conditions and thermomechanical properties of blends between wool and feather keratin and some polymers, especially PP and PE [103]. 

The preparation of blends between keratin and polypropylene (PP) has been described by many studies, mostly as a more biodegradable partial replacement for PP within the composites, where the main keratin source of choice was feather (β) keratin. A common trait among many studies was the use of coupling or interphase agents to increase PP-keratin interphase adhesion. Bullions et al. [104] produced composite panels made of feather keratin fibre (30 wt. %), kraft pulp fibre (30 wt. %), polypropylene (PP) and maleic anhydride modified polypropylene MAPP (40 wt. %). The composites were prepared via compression moulding from multiple plies of nonwoven, fabric-like prepreg manufactured with wetlay papermaking equipment, avoiding the higher temperatures of melt-mixing. They reported that the best mechanical properties were observed for the composition with 30 wt. % feather fibre, 30 wt. % pulp fibre, 8 wt. % MAPP and 32 wt. % PP, demonstrating that the mechanical properties improved proportionally with increasing MAPP content due to fibre–matrix interphase improvement. The same effect was observed by Barone and Gregoire [105], who described that MAPP (>4 wt. %) enhanced the stress at break and increased the amount of transcrystallinity, both as probable effects of the increased interphase adhesion between the feather keratin and the PP/MAPP matrix. The same authors used chicken feather fibres of a similar diameter but varying aspect ratio to melt mixing with low density polyethylene (LDPE), observing a decrease in density and an increase in elastic modulus and yield stress in the composites with a wide range of fibre loading [106]. 

Using a different keratin source, Kim and Bhattacharyya incorporated wool together with other additives such as ammonium polyphosphate (APP), talc and MAPP. The authors produced composite sheets with PP via melt-mixing using a twin-screw extruder followed by moulding with a single-screw extruder equipped with a flat die (170–180 °C) [107]. They evaluated the effective char formation to produce fire retardant composites, where, with a 30 wt. % wool and 15 wt. % APP addition (lower than usual fire-retardant applications), they achieved a direct self-extinguishing composite (V-0 rating). The thermal and mechanical properties were also improved by increasing wool–PP interphase adhesion with adding 2 wt. % of MAPP [107].

Instead of modifying the polymer matrix, Huda et al. [108] prepared PP-poultry feather keratin (70/30 wt. %) composite materials via melt extrusion, pre-treating the feathers with sodium hydroxide or 10% maleinized polybutadiene rubber (MPBR, impact modifier) or 3-aminopropyltriethoxysilane (APS) coupling agent (5 wt. % in relation to feather), for improving the interphase adhesion. Both the addition of feather keratin and all the interphase treatments improved the mechanical properties of the composites (flexural and tensile moduli and impact strength) in relation to neat PP. Similarly, Wang et al. [109] used Na_2_S_2_O_5_ modified duck and chicken feather fibres and their association with polypropylene (PP) (50:50) to produce effective melt-blown filter cartridges for Pb^2+^ adsorption.

Furthermore, Rivera-Armenta reported the direct melt-extrusion preparation of PP/keratin composites, without PP or feather modification, presenting good dispersion and compatibility by using a recycled PP matrix and chicken feather quills [110]. The composites presented enhanced storage modulus (especially at low feather quill loadings), thermal stability (especially at high quill content), and a decreased density.

Interestingly, using a completely different approach, Jain et al. [111] reported the graft copolymerization of powdered chicken feather with styrene monomer, without isolating the protein keratin or adding any polymerization initiator. They observed that the keratin acts as a support and initiator for the reaction, which only happened with the application of surfactant (sodium dodecyl sulfate, SDS), most likely by avoiding agglomeration and consequently, the inaccessibility of the fundamental functional groups.

#### 7.1.2. Polyethylene Glycol (PEG) and Polyethylene Oxide (PEO)

Polyethylene glycol (PEG) and polyethylene oxide (PEO) are polymers with the same chemical composition, but often referred distinctively by their molecular weight (*M*_w_), where PEG is correlated to *M*_w_ < 100,000 [112]. They are hydrophilic polymers obtained by ethylene oxide polymerization (thus consisting of a repeating unit of –[O–CH_2_–CH_2_]–), which are nontoxic and FDA-approved for use as carriers in different foods, cosmetics and pharmaceutical formulations. PEO/PEG presents an inherent ability to attach a variety of reactive functional groups to their terminal sites, making them especially suitable as cross-linking agents or molecular spacers. For that reason the term PEGylation has been coined, referring to the covalent grafting of a PEG derivative onto molecules, improving the water solubility and biocompatibility, which is especially useful for drug development [113,114].

The association of keratin with PEO and PEG has been mainly exploited for coating, fibre casting and hydrogel preparations, where, contrarily to polyolefins, the main sources of choice were wool and hair (α) keratin.

Tonin et al. [95] produced a series of studies, initially preparing blends of poly(ethylene oxide) (PEO) and wool keratin via solvent casting. They added the PEO into aqueous keratin solutions prepared by keratin extraction with urea, m-bisulphite and sodium dodecyl sulphate (SDS). The authors observed a melting point and a related enthalpy decrease with the increase of the keratin amount in the casted blends, indicating that keratin hinders the PEO crystallization process during solvent evaporation, and also that PEO seems to interfere with the keratin self-assembling, giving a different thermal behaviour to the protein. Interestingly, they also observed that blends with similar amounts of PEO and keratin tended to stabilize the β-sheet conformations, whereas, with larger amounts of PEO, the α-helix conformations were favoured. Later on, they studied the rheology of these wool keratin/PEO aqueous solutions, which displayed non-Newtonian flow behaviour, with strong shear thinning and a higher intrinsic viscosity than the neat keratin and PEO, suggesting a good interaction [115,116]. Then, they applied the solutions to produce electrospun wool keratin/PEO nanofibres, obtaining defect-free fibres with keratin amounts up to ~70 wt. %. The electrospinning process seems to hinder the natural self-assembly of S-sulfo keratin, leading to the formation of a less complex protein conformation, however, it also disrupts the keratin α-helix structure, consequently producing poor mechanical properties, especially in the keratin-rich fibres formed [116,117]. Similarly, Zhang et al. [118] prepared wool keratin/PEO nanofibrous membranes using keratin extracted from decolorized wool waste, via electrospinning. Initially, they studied the best discoloration approach to conserve the keratin structure, obtaining about 94.2% (in 210 min) discoloration using a catalytic oxidation with iron phthalocyanine, H_2_O_2_ at pH 10 and still allowing good structure stability. Then, PEO was added to improve the spinnability of the extracted wool keratin, where the nanofibres diameter increased proportionally with the increase in the PEO ratio, producing 546 ± 312 nm-thick fibres for 70/30 wt. % keratin/PEO membranes.

Moreover, Fan et al. [119] went a step further and prepared a water insoluble human-hair keratin/PEO (90/10 wt. %) blend nanofiber mat with high content of keratin via a two-step crosslinking process. A primary crosslinking process with ethylene glycol diglycidyl ether (EGDE) was applied for biocompatibility, followed by a secondary oxidative crosslinking process (in pure oxygen atmosphere) to improve the hydrophobicity of the electrospun keratin/PEO nanofibers. The authors reported that while the primary crosslinking improved electrospinnability, the secondary crosslinking gave hydrophobicity to the nanofiber. This produced a water-tolerant keratin/PEO blend nanofiber mat with a high keratin content, with improved crystallinity and thermal resistance, while still maintaining good cell adhesion, proliferation and growth.

Stojanovic et al. [120] took advantage of the previously described good interactions between PEO and poultry feather keratin to functionalize graphene with PEO using an ultrasound method and further mix it with poultry feather keratin to obtain nanocomposites via solvent casting. The authors describe that in a keratin/PEO (90/10) blend, increases in storage modulus (92% from DMA), reduced modulus (155%) and hardness (99% from nanoindentation) were inferred with the addition of only 0.3 wt. % of PEO functionalized graphene. They attributed the reinforcement to crystallinity changes and the effective load transfer between the reinforcing and matrix phases. 

Most recently, Yue et al. [121] prepared photo-cross-linkable human hair keratin (43 wt. %)-polyethylene glycol (PEG, 57wt. %) hydrogels using thiol-norbornene “click” chemistry, by producing free thiol groups on keratin and introducing norbornene groups to the PEG crosslinker. By using a photoinitiator (Eosin Y), the reaction, in stoichiometric ratio, could be activated by short exposition to visible light. The hydrogels displayed tuneable mechanical properties (up to 45 kPa compressive modulus) and long-term stability in buffer solutions and cell culture media. The keratin-PEG hydrogels were tested as cell culture substrates in two-dimensional surface seeding and three-dimensional cell encapsulation, demonstrating excellent cytocompatibility to support the fibroblast cells adhesion, growth and proliferation. The authors also demonstrated that the photo-activated crosslinking of the hydrogels allows micro-patterning and wet spinning to fabricate cell-laden tissue constructs with different architectures [121].

#### 7.1.3. Poly(ethylene imide) (PEI)

Poly(ethylene imine) (PEI) is an amine based polymer with –[CH_2_CH_2_NH]– repeating groups. It can be found in the linear form containing all secondary amines, in partially branched structures containing primary, secondary and tertiary amino groups, and in totally branched dendrimeric forms. PEI is produced at the industrial scale as a valuable cationic polyamine with uses ranging from a drug/gene carrier to a wood-adhesive component [122].

The association of keratin with PEI was only briefly explored for hair cosmetic application and to the best of our knowledge, by only one group. Kuzuhara and Hori used PEI to improve the colourability of human hair keratin fibres at low temperatures, by applying a pre-treatment with PEI prior to the application of an acid dye, where PEI acts as a counterion, considerably increasing the colouring speed [123]. They also performed an optical microscopy investigation on the penetration of PEI and Orange II dye into bleached human hair, concluding that PEI penetrates the hair cortex region, while the penetration of orange II into human hair increased proportionally with the increase of PEI treatment time and decreasing its molecular weight [123]. Then, they also observed that the same colouring process was improved with the addition of urea, by accelerating and increasing the length of penetration of PEI into the human hair [124]. Later on, they developed a method to more precisely investigate this diffusion behaviour of PEI into human hair keratin fibres, using cross-sectional samples of bleached white human hair treated with PEI. The post-treated cross-sectioned hair samples were dyed with Orange II and scanned with a microspectrophotometer at a 487 nm wavelength (ʎ_max_ of Orange II). The authors observed that the PEI diffusion coefficient is independent of the concentration and recorded a value of 10^−10^ cm^2^/s, for a PEI with *M*_n_ = 300 and 600 at pH 11.1. Moreover, they observed that the addition of urea accelerates the PEI penetration, outputting a twice higher permeation value for PEI (*M*_n_ = 600) [125].

#### 7.1.4. Polyacrylates, Polyacrylonitrile (PAN) and Polyacrylamide (PAM)

Polyacrylates are a broad group of polymers derived from acrylic acid, presenting a general chemical formula –[CH_2_CRCO_2_R’]–, yielding a series of highly transparent and elastic resins with good impact toughness used in vast applications. The different types of polyacrylates produced include the well known polymethylacrylate (PMA), polyethylacrylate (PEA), and polymethylmethacrylate (PMMA), with applications ranging from textiles and cosmetics to adhesives and paints [126]; and the poly(2-hydroxyethyl methacrylate) (pHEMA), as the first polymer matrix for soft contact lenses [127]. The substitution of the carboxylic acid group by a nitrile allows the polymerization of the acrylonitrile formed into polyacrylonitrile (PAN). PAN has a general chemical formula –[CH_2_CH(CN)]–, and since the 1950s, has been among the major precursors for synthetic fibres together with nylon and polyester, with about 2.73 million tons/year produced worldwide [128]. PAN is almost entirely produced as staple fibre, with the major use in bulky fabrics as an alternative to wool [129], especially because its fibres can also get the crimp structure like wool by using a bicomponent spinning process in fibre preparation [130]. In fact, PAN is not manufactured in its pure form and typically consists of 89–90% acrylonitrile, 4–10% non-ionic co-monomer (e.g., vinyl acetate) and 1% ionic co-monomer containing a sulfo (SO_3_H) or sulfonate (OSO_3_H) group [128]. In addition to allowing the polymerization of polyacrylamide (PAM) (–CH_2_CHCONH_2_–), the acrylamide is a common co-monomer for copolymerization with acrylates, in particular, adding an ionic character to the polymer matrix. PAM copolymers with acrylic acid and its sodium salts are often used in waste water treatment, but also have many other applications as, e.g., soil conditioner, absorbent, oil recovery and a thickening agent, flocculating and suspending agent, and lubricant [131].

The associations of acrylates, acrylonitrile and acrylamide with keratin have been studied for a long time, especially due to the textile industry’s interest in grafting onto wool keratin fibres (via thiol groups) [132] for affecting the water adsorption [133,134], mechanical properties [135] and evaluating the polymerization conditions [136] and the influence in the original fibre crystalline structure [137,138].

Concerning polyacrylates, Elangovan and Saccubai observed that the graft copolymerization of methyl methacrylate (MMA) onto the wool surface improves the acid/alkali resistances and the dye uptake, also increasing the wool’s tensile strength [139] and thermal properties [140]. Xu et al. [141] studied the graft polymerization of hydroxyethyl methacrylate (HEMA) onto woollen fabrics by microwave irradiation, yielding a much higher graft add-on by improving the monomer’s reactivity in comparison to conventional heating. They observed that the moisture regain decreases, while the max load and strain at max load increase, with increasing grafting [141]. Similar systems were prepared by Meng [142] and Freddi et al. [143], for the graft copolymerization of butyl methacrylate (BMA) and benzyl methacrylate (BzMA) onto wool fibres. They also observed that tensile strength increases with grafting, together with decreasing the elastic deformation for high modification degrees (>25% for BMA and >45% for BzMA) and decreasing the moisture retention and the molecular orientation (as seen by birefringence). 

Using cow hair keratin (10–50 wt. %) and acrylic resin (acrylic acid, butyl acrylate and methyl methacrylate; 50–90 wt. %), Zhang, Zhang and Shan [144] prepared a sound-insulating film. The formulation with 30 wt. % keratin produced equivalent sound-insulation to asphalt- or rubber-based insulators and superior to polyurethane foam-based insulators for a 20–20,000 Hz frequency range [144].

Using another keratin source, Castaño et al. modified the keratin fibres from chicken feathers through graft copolymerization with methyl methacrylate in an aqueous medium, using a KMnO_4_/malic acid redox system, resulting in slightly improved thermal properties of the PMMA-grafted feathers [145]. Similarly, Yang et al. grafted chicken feather fibres with methyl, ethyl, butyl, and hexyl methacrylates (MMA, EMA, BMA, and HMA, respectively), and produced transparent films with high humidity stability and tuneable tensile properties, and with stresses at break up to 7.0 MPa (MMA) and elongations up to 45.5% (HMA) [146]. More recently, Jain et al. [111] also reported the graft copolymerization of powdered chicken feather with methyl methacrylate (MMA) and glycidyl methacrylate (GMA) monomers. However, the authors performed the synthesis without isolating the protein keratin and only applying a surfactant (sodium dodecyl sulfate, SDS) without adding any free radical initiator, indicating a dual function of the keratin (catalyst/initiator and support matrix). The authors also observed a mandatory application of SDS for the reaction to happen, indicating the need for availability of the protein active sites that could be disturbed by agglomeration [111].

Concerning the association between keratin and PAM or PAN, Schaller et al. [147] described the preparation of composite membranes composed of Merino wool keratin and PAN, by graft polymerization of acrylonitrile (AN) onto a soluble keratin derivative. The authors, unfortunately, did not present any water adsorption data or thermomechanical characterization of the obtained composites [147]. On the other hand, Samal et al. performed the graft polymerization of acrylamide (AM) onto wool fibres and observed water adsorption, and the mechanical properties (both max load and strain) increased proportionally the grafting, whilst thermal properties had the opposite behaviour (proportional decrease with increasing grafting) [148].

More specific details on the mechanism of graft polymerization onto wool fibres and its effect on the structure, mechanical and thermal properties can be found in another review article by Shavandi and Ali [149]. 

#### 7.1.5. Polyvinyl Chloride (PVC)

Polyvinyl chloride (PVC), with a monomeric repeating unit –[CH_2_CHCl]–, is among the six majorly consumed plastics in Europe and contributed to about 61 million tons worldwide production in 2013 alone, with 38.5 million tons consumed and an estimation of about 3.2%/year increase until 2021 [150]. In addition to having been associated with toxicity and serious health issues for a long time [151], due to its cost-effectiveness and versatility, PVC is used in water, drainage and sewage pipes, and many other construction-related applications and extruded/injected parts [152], such as the vinyl resin-based phonograph record [153].

Poly(vinyl chloride) (PVC) blends with keratin have very poorly been explored in the literature, as only Rivera-Armenta et al. prepared PVC/chicken feather quill blends by melt-mixing [154] and Sharif et al. prepared various PVC/poultry feather keratin blends via a solution blending using *N*,*N*-dimethylformamide as solvent. The authors attribute dthe blend miscibility to interactions between carbonyl groups of the keratin structure and hydrogens geminal to the chlorine in the PVC, where increasing the keratin content resulted in enhanced blend miscibility. They also observed that the blends’ thermal stability increased with the feather keratin content [155].

#### 7.1.6. Polyvinyl Alcohol (PVOH)

Polyvinyl alcohol (PVOH), with an idealized chemical formula of the repeating unit –[CH_2_CHOH]–, is a linear synthetic polymer presenting good chemical resistance, water solubility, biocompatibility and biodegradability. PVOH is not a direct polymerization product of its structural monomer (i.e., vinyl alcohol), due to its unstable nature, but it is produced via vinyl acetate polymerization followed by the controlled partial alkaline hydrolysis (saponification) of polyvinyl acetate [156]. It is commonly used as an industrial product, especially in paper products manufacturing and textile industries. Moreover, PVOH is an FDA approved polymer, thus also often used in the food packaging industry as a gas/vapor barrier in food packaging (for close contact with food products), as a coating agent for pharmaceutical and dietary supplement products, and in medical devices [157,158]. 

Similarly to PEO and PEG, systems mixing polyvinyl alcohol (PVOH) and keratin have mainly been reported for the preparation of fibres or casted films, always presenting the use of plasticizers or coupling/crosslinking agents to avoid phase separation. 

Concerning fibre spinning processes, Katoh et al. [159] prepared PVOH blend fibres with 13–46 wt. % of (spray dried) sulfonated wool keratin by wet-spinning, with dehydration of an aqueous solution of the blend in a coagulation bath of sodium sulphate–saturated solution followed by drawing and thermal treatment at 195 °C for 10 min. The blend fibres containing up to 30 wt. % keratin displayed higher tenacity than wool and better waterproof characteristics than PVOH fibres, which was attributed to the crosslinking of disulphide bonds among keratin molecules during the heat treatment. The formed fibres were further used for adsorbing heavy metal and toxic gas, showing better efficacy to adsorb Ag^+^ and formaldehyde gas than PVOH [159]. More recently, Liu et al. [160] also prepared wool keratin (5–25 wt. %)/PVOH (75–95 wt. %) blend-fibres (D~110 μm) by wet-spinning, presenting increased thermal stability by increasing the keratin content (up to *T*_d5%_ ~230 °C with 25 wt. % keratin). On the other hand, the best mechanical properties were obtained at 5 wt. % keratin and decreased with increasing keratin content, including a sharp decline in the tensile properties at keratin contents above 15% [160].

Using solubilized chicken feather keratin in aqueous alkaline conditions, Wu et al. [161] mixed the keratin in an aqueous PVOH/citric acid solution for electrospinning keratin (10–30 wt. %)/PVOH (90–70 wt. %) nanofibers. The addition of 20% keratin to PVOH decreased the viscosity of the solutions, leading to a reduction in the spun fibre diameter from 565 nm to 274 nm, while larger keratin amounts resulted in beads-on-fibre morphology. The larger surface area of the thinner fibres, together with the higher keratin content, also promoted fibroblasts proliferation after 14 days. Moreover, the nanofibers crosslinking with citric acid fixed the morphology and pore structure even in the presence of water [161]. Moreover, Ding et al. [162] prepared a three-component chicken feather keratin/Polyvinyl alcohol (PVOH)/PEO (20/56/24) nanofibre membrane by electrospinning, followed by the application of a vapour-assisted crosslinking with citric acid or glyoxal. The method consists of exposing the already-prepared membrane to a large amount of vapour of the crosslinker, produced by heating at 60 °C. The authors observed that the method was more effective with citric acid than glyoxal. After treatment, the average nanofibre diameter increased from 223 ± 36 nm (non-crosslinked) to 342 ± 58 nm (citric acid-crosslinking) and 304 ± 55 (glyoxal-crosslinking) nm. Both treatments implied significant improvements in the membranes’ thermal stability and water resistance, and especially to the mechanical properties (tensile strength 4.5 times and elongation at break 3.7 times higher for the citric acid-treated membrane)[162]. Finally, Fathima and Kadirvelu [163] studied goat hair keratin extraction using five different hydrolysis methods/agents; namely, sulphitolysis, β-mercaptoethanol, ionic liquid, thioglycolic acid and alkali; determining the functional groups available and the structural effect inflicted (self-assembly) when blended to PVOH (8 wt. %) to spinning fibres. The authors observed that only the sulphitolysis and β-mercaptoethanol based mat showed evident change correlating the structure–property relationship. The sulphitolysis implied a high tensile strength (around 5.5 MPa) and a low mass transport resistance, while β-mercaptoethanol implied a higher melting temperature (around 290 °C) and biocompatibility [163].

Concerning film casting preparation of PVOH/keratin blends, El-Sayed et al. [164] dissolved keratin from different sources (animal wool, camel, hair, human hair and chicken feather) in various basic media (NaOH, LiOH, Sr(OH)_2_ and Ba(OH)_2_) forming keratin (67 wt. %)/PVOH (33 wt. %) composites via casting, using glycerol as plasticizer. The authors observed a clear distinction in the solutions’ apparent viscosity by varying the keratin source, also reporting that the tensile strengths of all the keratin/PVOH films are lower than both neat PVOH and neat keratin, while the elongations at break of all films are higher than that of neat PVOH [164]. In addition, a series of solution-casted blend films based on chicken feather keratin (80–100 wt. %), PVOH (0–20 wt. %), and dialdehyde starch (DAS, 0–15 wt. %) as crosslinker, were prepared by Yin et al. [165]. The blends presented compatibility, with a single glass transition and melting temperature and increases in tensile strength, elongation at break and decomposition temperature were also observed with increasing PVOH content. Moreover, the increase of the DAS amount caused tensile strength, thermal resistance and water resistance to increase, while elongation and water vapour permeability decreased, indicating an increase in crosslink density [165]. Then, the authors further characterized the same systems considering potential drug release applications. They observed that the crosslinking with DAS decreased the films crystallinity and their total water soluble mass below 35% at 37 °C. Furthermore, they applied the films as vehicles to release Rhodamine B dye (as a model drug) and observed that the release rates decreased proportionally with increases in the amount of DAS, allowing the controlled release in function of the crosslinking [166]. Furthermore, Chen et al. [167] prepared similar chicken feather keratin/PVOH blend films compatibilized by tris(hydroxymethyl) aminomethane (Tris) via solution casting. The authors reported the formation of a partially crystalline phase separated system with the components mainly interacting via H-bonding. They observed that by increasing the PVA content, the elongation at break, hydrophilicity and oxygen barrier properties were enhanced, while the elastic modulus and water vapour barrier properties decreased. On the other hand, increasing the amount of Tris increased the tensile strength, elongation at break and oxygen barrier properties, while the contact angle decreased, with Tris playing the role of plasticizer in the blend [167]. 

#### 7.1.7. Polyamide-6 (PA6)

Polyamide 6 (PA6), also widely known as Nylon 6, was originally synthesized in the late 1800s by ring-opening polymerization of *ε*-caprolactam or self-condensation of *ε*-aminocaproic acid, presenting a repeating unity –[C_6_H_11_NO]–. PA6 entered the market for the first time in Germany in the late 1930s, only a few years after the launch of the Nylon 6,6 by DuPont Company, as one of the first truly synthetic fibres [168,169]. To date, it is one of the most used types of aliphatic polyamide, mainly applied in fibres, films, and as injection-moulded engineering plastic, being used to produce everything from umbrellas, stockings, camping tents and guitar strings, to children’s toys and medical implants. One of the main reasons for PA6’s vast application range is its excellent thermomechanical properties, including its high modulus even above the glass transition temperature. However, PA6 is highly hygroscopic and the absorbed water has a large influence on its properties [170].

Such as in the cases of PEO, PEG and PVOH, the blends of PA6 with keratin were mainly explored via the preparation of fibres or casted films. Concerning fibre formation, Aluigi et al. [171] prepared mats of randomly oriented nanosized filaments by electrospinning Merino wool keratin/PA6 blends in formic acid, forming nanofibres with diameters between 230 and 130 nm. They reported that the nanofibres are effective Cu^2+^ ion adsorbents (superior to commercial activated carbon) and the effectiveness increases with an increase in the specific surface area of the nanofibre mats, where 50, 70 and 90 wt. % keratin in the composition adsorb 61.7, 90 and 103.5 mg/g, respectively [171]. More recently, the same group demonstrated the preparation of similar systems by electrospinning of the immiscible dispersions of keratin and PA6 [172]. They obtained homogeneous blends that they attributed to fast solvent evaporation (kinetic effects prevail over the thermodynamic ones), as opposed to the solvent casting technique that forms a defined segregated morphology. Keratin nanodomains varied from 100 to 250 nm, depending mainly of the keratin content, where the percentage of keratin is negatively correlated to increasing diameters while viscosity and conductivity are positively correlated to increasing diameters (voltage and flux influence was negligible). The authors also observed that the keratin presence seems to hinder the formation of α-crystallites of PA6, and keratin/PA6 blends form an unusual crystalline configuration in the nanofibers [172].

Sharif et al. [173] prepared solution-casted poultry feather keratin/PA6 blend films and investigated the individual roles of the polymers in the blends formed. In contrast to the macrophase separation described by Aluigi et al. [171] when solvent-casting wool keratin/PA6 blends, the authors observed a tendency for nanoscale phase separation between PA6 and feather keratin. The evaluation of the blends’ surface topography and roughness by AFM also revealed that the keratin-rich blends had coarser surfaces than PA6-rich ones, while amplitude–phase–distance measurements revealed that the blend phase inversion occurs at a 40 wt. % feather due to the significant difference between the molecular weights of the blend constituents. Using nanoindentation experiments, they also observed that PA6 was responsible for improving the blend elastic modulus and stiffness, while keratin provided higher pull-off force and work of adhesion for the blends [173].

#### 7.1.8. ε-Polycaprolactone (PCL)

*ε*-Polycaprolactone (PCL) was first obtained in the 1930s by thermal treatment of ε-caprolactone, yielding a polymer composed of hexanoate repeat units (–[C_6_H_10_O_2_]–), included in the class of aliphatic polyesters [174]. To date, PCL is mainly synthesized by ionic and metal catalysed ring-opening polymerization of ε-caprolactone and has recently returned once again as one of the most explored polymers, especially for its peculiar mechanical properties, large miscibility range with other polymers and biodegradability. It has also been certified as an FDA-approved (United Stated of America) and EC registered mark (Europe) for use in a large number of drug-delivery and medical devices. More recently, the PCL biodegradation associated to the superior rheological properties (including easy processability) has also attracted interest for the design of biodegradable devices [175,176].

The associated use of keratin and PCL was mainly explored for fibre casting via the electrospinning technique, especially for cell proliferation scaffolds and supports with controlled mechanical properties, where the α-keratin sources were wool and hair, such as the case of Li et al., [177] that prepared nanonets of wool keratin and poly(ε-caprolactone) (PCL) via one-step electrospinning with formic acid. A dual structure was formed consisting of randomly oriented D = 299–624 nm nanofibers and dense spider-web-like D = 25 ± 5 nm nanonets, with nanonet formation only at large keratin amounts (≥25 wt. %). The keratin addition to the structure formed hydrophilic nanonets decreasing the water contact angles 20–50 degrees; however, the mechanical properties also suffered a sharp decline in comparison to the neat PCL nanofibers [177]. Later on, the same group prepared similar electrospun blends with different ratios of wool keratin and PCL for accessing their morphology, biodegradation degree (in phosphate buffer saline, PBS) and cell proliferation. They observe that the increased hydrophilicity by the addition keratin to the PCL also proportionally promoted faster biodegradation (weight loss 28% in 50 days for keratin/PCL = 60/40) and stimulated a more significant level of in vitro mouse fibroblast cell adhesion and cell viability [178].

Similarly to the wool fibre-based materials, Battarai et al. [179] blended human hair keratin with PCL in different ratios by electrospinning technique, forming nanofibrous membranes. The authors reported that PCL/keratin blends containing up to 30 wt. % keratin showed uniform fibre morphology, structural integrity, suitable mechanical properties and cellular compatibility. Subsequently, the same group included magnesium oxide (MgO) to the human hair keratin-PCL blends, forming uniform ternary composites nanofibers, D = 0.2–2.2 μm, via electrospinning. They observed that both PCL/keratin and PCL/MgO blending cause a considerable decrease in the original PCL mechanical properties. However, the PCL/keratin/MgO composite avoided the detrimental effect, presenting ultimate tensile strength and Young’s modulus up to 3 and 5.5 MPa, respectively [180]. In addition, Loo et al. [181] electrospun hair keratin (30 wt. %) and PCL (70 wt. %), crosslinked with glutaraldehyde and further coated with calcium phosphate to prepared osteoconductive composite scaffolds. They observed the formation of scaffolds with 2.66 µm pores, presenting a homogeneous calcium phosphate coating, producing a high proliferation of human mesenchymal stem cells, and good tensile strength (16.53 MPa), strain at break (153%), elastic modulus (25.92 MPa).

#### 7.1.9. Polylactic Acid (PLA)

Polylactic acid (PLA) was discovered by Carothers in 1932 by heating the lactic acid under vacuum while removing the condensed water, yielding a low molecular weight thermoplastic aliphatic polyester with the repeating units –[C_3_H_4_O_2_]– [182]. Later on, using ring-opening polymerization of lactide, the production of higher molecular weight PLA was reached, nowadays the most often used industrial approach [183]. PLA is considered a bioplastic, since its precursors are derived from fermentative processes of renewable biomass, typically from plant starch from corn, cassava, sugarcane or sugar beet pulp, also presenting very low, or even negative, CO_2_ residual emissions. It is also immunologically inert, busting its broad application in the medical field, especially in wound healing, medical implants and prosthetics [182]. In 2010, the PLA had the second highest consumption volume of any bioplastic in the world, and the demand is increasing since it is the most extensively applied polymer to fused deposition modelling (FDM) 3D printing technique [184].

Concerning the preparation of keratin-PLA associations, varied keratin sources have been applied, using mainly solvent casting, electrospinning and melt-compounding techniques. Puglia et al. [185] used three different keratin sources (Merino wool, Brown Alpaca fibres and commercial hydrolysed keratins) as fillers in PLLA based biocomposites via solvent casting in chloroform. The biocomposites presented a phase adhesion strictly dependent on the keratin source, consequently affecting also the surface topology, transparency, wettability, thermal and mechanical properties, and inducing different stem cell organizations on the substrate. The authors highlighted the possibility of mechanical properties control and different stem cell organizations on the substrates by simply changing the keratin source. Aiming to produce scaffolds for favourable distribution of biological molecules and cell ingrowth, the same authors used the same keratin sources (Merino wool and Brown Alpaca fibres) to produce biocompatible PLLA/keratin tridimensional scaffolds via two methods, namely solvent casting followed by porogen (paraffin) particulate leaching, and a thermally induced phase-separation process. The authors reported that the scaffolds porosity and architecture were highly sensitive to the porogen content and solvent/non-solvent ratio, allowing the formation of a variety of microcellular and porous foam morphologies [186]. Also using wool keratin, Li et al. [187] produced hydroxyapatite (HA) in situ into a wool keratin solution and electrospun together with poly(L-lactic) acid (PLLA), forming a fibrous membrane. The solution presented good electrospinnability and formed membranes that induced significant bone formation in comparison to neat electrospun PLLA, which the authors attribute the strong interaction between the keratin functional groups and the Ca^2+^ from HA.

Huda et al. [108] prepared PLA/poultry feather keratin (70/30) composite materials by melt extrusion, with prior treatment of the feathers with sodium hydroxide or 10% maleinized polybutadiene rubber (impact modifier) or 3-aminopropyltriethoxysilane (APS) coupling agent (5 wt. % in relation to feather), for improving the interphase adhesion. All the treatments, including the sole addition of feather keratin, improved interphase adhesion during extrusion, improving the mechanical properties (>9 GPa flexural and >4.5 GPa tensile moduli) in relation to neat PLA (~4.5 GPa flexural and ~3.5 GPa tensile moduli) [108]. Similarly, Spiridon et al. [188] observed that the addition of feather keratin fibres (2–4 wt. %) improved the elastic modulus (3.3 GPa), tensile strength (65.1 MPa), impact strength (11.1 KJ/m^2^) and thermal stability of PLA matrix, also decreasing the detrimental effect in impact strength when adding chitosan to PLA, in the preparation of PLA/chitosan (70/30 wt. %) composites. However, the addition of keratin decelerated the PLA/chitosan degradation, as seen by the application of accelerated weathering. The authors also observed a selective degradation of the amorphous part of the composites and chain cleavage by UV exposure was the main degradation process [188]. On the other hand, Aranberri et al. [189] produced materials based on PLA, polybutyrate adipate terephthalate (PBAT) and a PLA/thermoplastic copolymer blend, with much higher loadings of chicken feather fibres (50 and 60 wt. %), manufactured with a torque rheometer. Independently of the polymer association, the formed composites presented a lower density, increased water adsorption and thermal insulating properties. However, the thermal stability, tensile strength and elongation-at-break were negatively affected. The elastic modulus was dependent on the polymer matrix and the composites with PLA had the modulus practically unaltered, while the PBAT and the PLA/thermoplastic copolymer blend became stiffer with feather addition [189]. Moreover, Carrillo et al. [190] observed that the elastic modulus of PLA is not considerably affected by the feather content, while the tensile strength and the elongation decreases by up to 58% and 12%, respectively, for 25 vol. % chicken feather addition. However, these keratin/PLA composites still present better tensile properties than medium-density fibreboards and organic resin-bonded particleboards.

It also worth mentioning the work of Sanches-Olivares et al. [191] who prepared animal hair keratin fibre/PLA composites via melt compounding both with and without adding together a traditional flame retardant (aluminium trihydroxide, ATH). They observed that the addition of keratin fibre into PLA classifies it as V-2, and the combination of keratin fibre and ATH as V-0 flame retardant, by the UL94-V standards. Moreover, the addition of keratin fibre reduced the polymer matrix viscosity, consequently improving the composites processability.

#### 7.1.10. Polyhydroxyalkanoates (PHA)

Polyhydroxyalkanoates (PHA) are a family of natural and biodegradable polyhydroxyesters produced by bacterial fermentation of sugar or lipids under nutrient-limiting conditions with carbon excess. In contrast to other bio-based polymers, such as the PLA that is in vitro synthesized from a natural-based monomer, PHAs are fully synthesized in vivo [192]. The most representative examples of PHAs are the poly(3-hydroxybutirate) (PHB) and its hydroxyvalerate copolymer poly(3-hydroxybutyrate-co-3-hydroxyvalerate) (PHBV). The PHB was described for the first time in 1925 by Lemoigne, which discovered PHB insertions in Bacillus megaterium cells, and nowadays, its uses vary from biomedical applications to compostable bags and food packaging [193]. However, studies of PHAs blending with other natural polymers or fillers have been increasing, as a manner of remediating/compensating some of their properties flaws and the high cost of production [194].

The cytocompatibility of electrospun PHBV fibres has been shown to improve in association with keratin, as demonstrated by Kang et al., using a commercially acquired keratin (source not disclosed), with increased proliferation and attraction of the cells to electrospun PHBV fibres for wound dressing materials [195]. Similarly, Shen et al. [196] evaluated PHBV blends with collagen, gelatine and keratin, for the preparation of electrospun nanofibrous mats. All three proteins yielded enhanced cell compatibility to the blends; however, collagen promoted even better cytocompatibility than gelatine and keratin.

Lagaron et al. [197] published a series of studies about the association of PHBV with feather keratin. The authors initially developed composite materials based on a PHBV polymer, containing 12 mol. % hydroxyvalerate, and poultry feather keratin via melt compounding. The composite containing 1 wt. % keratin presented a good interphase interaction, causing increased mechanical performances and about 50% reduction in water, limonene, and oxygen permeability, in comparison to the neat matrix. On the other hand, they reported that the addition of keratin amounts larger than 10 wt. % was detrimental to most of the properties [197]. Later on, they prepared similar composites using two different PHBV polymers, containing 3 and 12 mol % hydroxyvalerate, using two different approaches: (i) the direct keratin-PHBV melt compounding or keratin pre-incorporation into an electrospun PHBV masterbatch with subsequent melt compounding with PHBV pellets; and (ii) a multilayer system by film (solution) casting of keratin followed by hydrophobization by coating with electrospun PHBV fibers. The authors observed that the amount of hydroxyvalerate in the PHBV grade influences the amount of keratin to be stabilized in the composite. The composites with incorporated keratin presented reduced water vapor (for both PHBV grades and approaches) and oxygen permeability (dependent on the PHBV grade). The keratin pre-incorporation method also improved the stretchability of the composites, while the multilayer approach produced hydrophobic surfaces (contact angle values >70°) [198].

#### 7.1.11. Thermoplastic Polyurethanes (TPU) and Polyurea-Uretanes (TPUU)

Thermoplastic polyurethanes (TPU) are block copolymers obtained by the reaction of polyol (ether-, ester-, and carbonate-based diols with M_w_ from 1000 to 3000 g/mol) with aliphatic or aromatic diisocyanates. They form alternating sequences of hard and soft segments, whereby the ratio’s variation determines the whole structure and properties of the TPUs. Consequently, a broad variety of TPUs can be obtained with small process variations, yielding polymer systems with modulable flexibility, mechanical strength, elasticity, good abrasion resistance and transparency [199]. Although currently, TPUs are mainly used in high-performance applications, such as adhesive, textile coating or impact modifier, they have also attracted attention with applications in FDM 3D printing, including for medical grade use [200]. Polyureas are important analogues of polyurethanes prepared by reacting diisocyanates and polyamines, and are typically used as protective coatings. Thus, the addition of polyamines to the TPU synthesis allows the preparation of thermoplastic polyurea-urethanes (TPUU) [201], where the urea linkages improve the intra- and intermolecular H-bonding and the mechanical properties [202,203].

Keratin-TPU associations have been prepared for applications such as scaffolds for cell growth/wound dressing films, foams and artificial skin, where mainly feather and hair have been used as the keratin source. 

Martínez-Hernández et al. [204] dissolved chicken feather keratin in a urea and 2- mercaptoethanol solution, and further incorporated it into a TPU matrix in two different ways: (i) by direct addition of the keratin solution and (ii) after dialyses treatment. They observed that the keratin was grafted in the TPU matrix, forming a keratin/TPU graft copolymer with a cellular morphology. The authors also observed that keratin/TPU membranes formed in the presence of salt (non-dialyzed) present groups and bonds not found in the dialyzed systems, and consequently, different properties, suggesting a role of the processes impurities in the grafting. The incorporation of the dialyzed keratin caused an increase while the non-dialyzed one caused a decrease in the thermal stability of the grafted copolymer. These systems also presented different thermal transition behaviours [204]. Differently, Wrześniewska-Tosik [205] prepared composites based on elastic polyurethane (EPUR) combined with milled poultry feathers (without prior keratin extraction) to produce foam materials. The addition of feathers to EPUR affected the foaming process, consequently decreasing the density, increasing the limiting oxygen index (decreasing combustibility and avoiding molten polymer droplet formation), and increasing the maximum degradation rate temperature of the resulting foams.

Ozkoc et al. [206] prepared porous composite scaffolds based on feather keratin fibre and TPU, via solution casting, combining salt leaching and thermally induced phase-separation methods. The authors observed a homogeneous morphology and highly porous structure with evenly distributed and interconnected pores, in which the storage and elastic moduli together with the strength of the composites increased with a keratin content up to 40 wt. % (larger keratin amounts were detrimental). However, the composite’s hydrophilicity was enhanced at high keratin contents (about 80%). Moreover, the cytotoxicity and biocompatibility were also increased, showing mouse fibroblast cell viabilities higher than 80% for all the scaffolds prepared. Similarly, Jones et al. [207] prepared chicken feather keratin/TPU composites via solvent casting, describing an effective interphase adhesion, with no agglomeration and an even distribution of fibres. The authors observed that the addition of feather fibres proportionally decreased the glass transition temperature, the thermal resistance and the recovery strain, but increased the elastic and storage moduli and the char yield after thermal decomposition. They attributed to 20% as an optimum volume fraction of feather fibres based on the composite’s elasticity. In agreement with Martínez-Hernández et al. [204], they also suggested the formation of chemical bonding between keratin and TPU, as observed by molecular modelling and FTIR [207].

Using a different keratin source, Shen et al. [208] prepared a nanofibrous mat for wound dressing combining TPU, human hair keratin, and silver nanoparticles (AgNPs). The authors initially extracted the keratin by modifying it with iodoacetic acid to obtain S-(carboxymethyl) keratin, which was then blended with TPU and electrospun, followed by in situ formation of AgNPs, resulting in antibacterial TPU/keratin/AgNP mats. The authors observed that the introduction of keratin promoted fibroblast cell proliferation, which was not weakened by the loading with AgNPs and resulted in good antibacterial properties. They performed an in vivo wound healing test and a histological examination, where the TPU/keratin/AgNP composite materials remarkably accelerate the wound recovery with very mild inflammatory responses. Recently, Kim et al. [209] observed that similar TPU systems containing 1 wt. % hair keratin display properties mimicking those of human skin, i.e., elastic modulus of 31.44 MPa, ultimate tensile strength of 18.52 MPa, coefficient of friction of 0.18, water contact angle of 85°, and very high toughness, similar to that of mammalian collagen fibrils (77.5 10^6^ J/m^3^). Thus, the authors applied extrusion-based melt-mixing method to these systems for preparing an artificial skin, reporting that the unique mechanical and tribological performances are a result of the formation of TPU-keratin H-bonding and the lubricating effect of cysteine-rich keratin during the melt-mixing [209].

Concerning the association between keratin and TPUU, only one work was found in the literature from Aranberri et al. [210] The authors studied two analogous TPUU elastomers prepared with two different diamine chain extenders (bis(4-aminophenyl) disulfide and bis(4-aminophenyl) methane, for the preparation of composites with high chicken feather fibres loadings (40–75 wt. %) in a torque rheometer and hot compression. Properties such as density, relative water absorption and relative thickness swelling were similar to both types of composites, however, systems prepared using the bis(4-aminophenyl) disulfide chain extender displayed the best interphase adhesion, consequently causing the best mechanical improvements (up to 7.5-fold higher tensile strength for 50/50 wt. % TPUU/feather fibres composite) [210].

### 7.2. Keratin Associations with Elastomers and Thermosets

**Figure 14:**
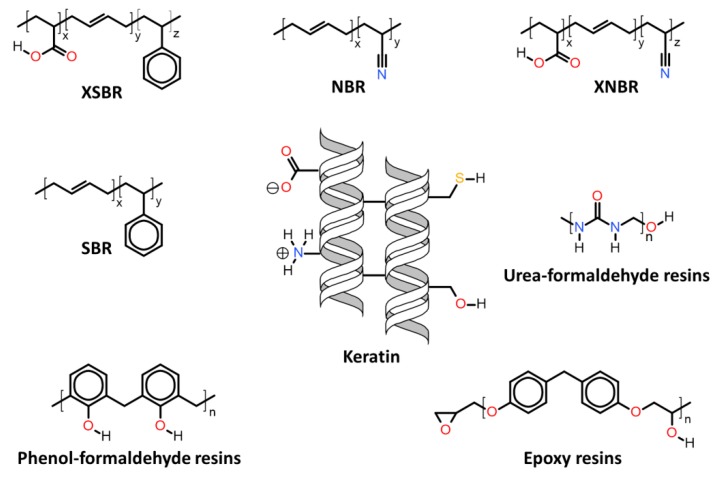
Schematic representation of the elastomers and thermoset polymers discussed, evidencing their generalized structures and available functionalities for interacting with keratin.

Thermoplastics/thermosets blends are complex systems since they naturally tend to a macrophase-separation, therefore requiring strict control of the phase behaviour, morphology and interfacial adhesion to convert these immiscible blends into crosslinked polymer materials [211]. However, once this hurdle is surpassed, they produce interesting systems due to their ability to partially fix the blend morphology, even when unstable, allowing a very broad set of properties that can be fixed together with the morphology [212]. Moreover, the previously described chemical toolset presented by keratin can allow it to participate in the crosslinking process and tightly binding it to the polymer network formed. The generalized structures of the polymers discussed and their different functionalities available are represented in Figure 14, and at the end of this section, Table 2 summarizes the main processes used and the thermomechanical properties achieved with the keratin/thermoset polymer blends.

#### 7.2.1. Butadiene Copolymer Rubbers

Polybutadiene-derived rubbers are the main components for tires production and additives for toughness improvement in thermoplastic polymers, due to their high resistance to wear and impact strength [213]. Copolymerization of butadiene, e.g., with styrene forming styrene-butadiene rubber (SBR) or with acrylonitrile forming nitrile-butadiene rubber (NBR), is generally used for improving the chemical resistance and producing elastomers that are resistant to oil and chemicals [214]. However, most butadiene rubber applications demand blending with inorganic fillers or other polymers to acquire enough mechanical properties [215], in which the application of biopolymers can improve their post-use biodegradability [216].

The application of keratin in association with butadiene copolymers for the production of fibre coatings or for increasing the rubber crosslinking efficiency was mainly associated to one group, namely Prochoń et al. [210] Initially, the authors prepared composites with cattle hair keratin, zinc oxide and carboxylated acrylonitrile-butadiene rubber (XNBR), describing that the addition of keratin increases the crosslinking density causing improvements in mechanical proprieties (such as tensile strength and hardness) and chemical resistance to fuel and oil. The authors ascribed these improvements to the formation of ionic bonds, and reinforcement in the covalent crosslinking since the keratin promotes mono- and disulfide bonds, which are more stable than the polysulfide bonds in standard vulcanizates. Moreover, they suggest that since the keratin filled composites have their water adsorption increased over time, also increasing electric conductance, they would be biodegradable after their use is expired (however, no biodegradability test was provided) [217]. They also observed similar results for systems with hair keratin and zinc oxide associated with styrene-butadiene rubber (SBR), describing improved resistance to thermooxidative aging, thermal and mechanical properties, and decreased flammability [218] and with nitrile-butadiene rubber and modified montmorillonite clay (MMC), observing increased water adsorption and thermal stability, and flammability decreased, proportionally, with increasing the keratin amount, while the mechanical properties were more dependent on the MMC amount [219]. Later on, the authors also produced a cellulosic–elastomeric material by coating cotton fibres with keratin and carboxylated styrene–butadiene latex (XSBL). They observed that the elastomeric coating increased the tensile strength and slightly increased glass transition temperature, also promoting different mechanisms of thermal decomposition, where the thermal decomposition residue was higher, in comparison to the neat cotton fabric [220].

Rivera-Armenta et al. prepared a styrene-butadiene/chicken feather via melt-blending with different reinforcing fillers such as zinc oxide [221], thermoplastic starch (TPS) and vinyl alcohol copolymer (EVOH) [222]. Similarly to the solution casting results previously discussed, they observed an improvement in the storage modulus and thermal stability of the composites by adding keratin, showing a general positive effect of keratin in the elastomeric systems, partially caused by its sulphur-containing segments.

#### 7.2.2. Epoxy Resins

Epoxy resins are thermosetting resins that allow their reticulation using a wide variety of crosslinkers, with their final properties directly dependent on the resin/crosslinker structure/functionality and resulting crosslinking density [223,224,225]. Although epoxy resins were already discovered in 1909 by Prileschajew [226], due to their excellent mechanical properties, high adhesiveness to many substrates, and good heat and chemical resistances, still today, they are among the major components in coatings and adhesives, and reinforced materials for industrial tools, aerospace industry, automotive industry, electronics and biomedical applications [227]. As a consequence, in 2011 alone, epoxy resins generated $5.5 billion dollars in revenue. However, 75% of their production is based on the diglycidyl ether of bisphenol A (DGEBA), being far behind the thermoplastic polymers industry in adapting to the new and growing bio-economy. Among the approaches studied to remediate this matter are the preparation of bio-based epoxy precursors, replacing the DGEBA pre-polymer [228]; the use of bio-based curing agents [229]; and blending with other biopolymers.

Concerning the latter, the association of keratin with epoxy resins was mainly explored for the preparation of composites via compression moulding and hand lay-up techniques using (β) keratin as a reinforcing (filler) phase.

Extensive work has been done by Wool’s group, where they fabricated composites with epoxy, chicken feather fibre, and E-glass fibres and investigated their properties for potential applications as printed circuit boards (PCB). The electrical resistivity of the feather fibre composites was two to four orders of magnitude higher than that of E-glass fibre composites, with the dielectric constant decreasing with an increasing fibre content. The composite with hybrid fibre (feather and E-glass fibres) presented a low dielectric constant of 3.6–4.2 and a loss tangent of 0.027 (similar to those of commercial PCB materials) [230]. Later on, they prepared chicken feather fibre reinforced epoxy composites, with 0 to 67 vol. % of feather fibres, compressed using a hot press and cured at 120 °C for 4 h to form 2 mm-thick composite panels. The authors observed that these composites present anisotropic thermal expansion behaviour, with negative values of coefficient of thermal expansion in the axial and positive values in the radial direction, and the application of feather fibres reduced the overall coefficient of thermal expansion and minimize the coefficient of thermal expansion mismatch [231]. The same group further observed that loading epoxy matrix with hybrid glass fibre/chicken feather fibres reduced a density up to 40% when compared with standard glass fibre reinforced composites. The feather fibre/epoxy composites displayed storage modulus of about 3.5 GPa and a flexural strength of about 50–80 MPa, while partially replacing feather fibres with E-glass fibre (hybrid fibre composite) can increase the modulus (13.4 GPa) and strength (about 310 MPa) [232].

Using a similar approach for different applications, Bessa et al. prepared chicken feather fibres/epoxy composites (with 60 to 80 vol. % feather fibres) via compression moulding at 120 °C, using 2 tons pressure for 6 min. They evaluated the thermo-acoustic properties of the composites and observed that the acoustic insulation was not very significant (up to 6.7 dB at 500 Hz). However, thermal resistance increased with an increase in the feather content, obtaining values up to 0.175 m^2^ K W^-1^ for the maximum feather loading (80 vol. %) [233]. Finally, Verma et al. applied alkali treated (NaOH) chicken feather fibre (1, 3, 5 and 7 wt. %) into carbon residuum (CR, obtained from crumb rubber, 0.5, 1, 1.5, 2, and 2.5 wt. %) fused with epoxy resin/triethylenetetramine (TETA) to form hybrid composites using the hand lay-up technique. The authors reported an optimum keratin content of 5 wt. % for impact test performance, and considerable improvement in tensile strength, flexural strength and impact strength with 1 wt. % CR addition [234].

Alternatively to the standard epoxy resins, Wool and co-authors also intensively explored the preparation of composites based on feather keratin and acrylated epoxidized soybean oil (AESO). In their initial studies, they applied 30 vol. % of chicken feather fibres into an AESO resin, producing a reinforced partially hollow composite, which due to the retained air presenting a dielectric constant k = 1.7–2.7 depending on the fibre volume fraction, values significantly lower than those of conventional silicon dioxide or polymer dielectric insulators. The incorporation of feather fibres in AESO also decreased the thermal expansion coefficient and enhanced the mechanical properties (100% increase of storage modulus, fracture toughness and flexural properties) [235]. They also produced composites with phthalated AESO (PAESO), chicken feather fibres and E-glass fibres, aiming to replace the application in printed circuit boards (PCB) of traditional E-glass fibre reinforced epoxy composites. The composites exhibited promising mechanical properties, dielectric constants, coefficients of thermal expansion and flammability characteristics, comparable to those of traditional composites applied to PCBs fabrication [236].

In addition to the direct application of feather keratin to composites, the same group also investigated the pyrolytic transformation of chicken feather fibres using a two-step method at 215 °C (24 h) and ~450 °C (1 h), resulting in the production of an active carbon-like microporous material (pore diameter < 1 nm) with narrow pore size distribution [237]. They described the pyrolytic transformation as disulphide bond cleavage followed by peptide crosslinking, which allowed tuning of the final network structure by controlling the debonding/crosslinking using different temperature profiles [238]. The reduced pore size of the pyrolyzed fibres allowed their application in hydrogen storage, adsorbing 0.4 and 1.5 wt. % excess hydrogen at room temperature and 77 K, respectively [239]. Then, the pyrolyzed fibres were also applied as reinforcing fillers in AESO and methacrylated lauric acid (MLAU) resins, producing mixtures with appropriate viscosity and thermal resistance for liquid moulding technique. A broad variety of mechanical performances (20–300 MPa storage moduli and 10–150 MPa tensile moduli) tuneable by the amount of pyrolyzed fibres applied (0–32wt. %), where the incorporation of 32 wt. % of feather fibres increased the storage and tensile moduli 15 times, being suitable for prospective applications in elastomeric materials or adhesives [240].

#### 7.2.3. Urea-Formaldehyde Resin

Urea-formaldehyde resin, synthesized via urea and formaldehyde (or metanal) polycondensation forming the repeating unit -[(O)CNHCH_2_NH]-, was first synthesized in 1884 by Hölzer and published by Tollens [241]. It is part of the amino resins, in which it constitutes about 80% of the global production of this class of resins [242], with applications from automotive tires and the paper industry to electrical and thermal insulation materials. Moreover, urea-formaldehyde resin is one the most important adhesives for the wood composite industry, used to prepare particleboards, plywood and medium density fibreboards (MDF) [243]. As for the case of epoxy resins, the high industrial demand for urea-formaldehyde resins makes the decrease in toxicity and increase in their sustainability quite appealing for economic and environmental reasons. Small modifications in these materials represent a considerable reduction of toxic industrial waste and formaldehyde emission during their lifetime [244]. 

Addressing this issue, Pang et al. modified urea-formaldehyde resin with feather keratin through copolymerization reactions, with an optimum urea/formaldehyde molar ratio of 1.3/1. They added 5 wt. % of keratin and reported that the keratin addition after the third feeding of urea is the best choice, producing a low toxicity modified urea-formaldehyde resin with reduced production costs [245]. Subsequently, Dim applied keratin-modified-urea-formaldehyde resin adhesive for bonding particleboards (80% wood chips, 11% resins and 9% moisture). The boards produced with keratin modified resin had an improved tensile (1.85 MPa), shear (0.98 MPa), compression (1.42 MPa), and bending strength (3.20 MPa), together with water resistance, overcoming the results with neat resin and meeting the minimum requirements of the Atapex standard [246].

#### 7.2.4. Phenol-Formaldehyde Resins

Phenol-formaldehyde resins, also known as phenolic resins, can be produced via two main pathways; i) formaldehyde/phenol (with ratio <1) reaction (novolacs) terminated by acid-catalysis, forming low Mw linear polymers (pre-polymers), which requires the application of a hardener to form thermosets; and ii) the base catalyzed formaldehyde/phenol (with ratio >1) reaction (resoles), creating a reactive phenoxide group that reacts with the formaldehyde to create a repetition unit -[(C_6_H_3_OH)–CH_2_]–. Phenolic resins were invented by Leo Baekeland, namely Bakelite, in 1907 [247]. Bakelite was the first truly synthetic resin exploited commercially and has been continuously used for over a century, although the total revenue of phenolic resins has not increased much since the 1950s due to the ever-growing availability of high-performance thermoplastics [248]. They are predominantly employed in laminated materials for the furniture, building, transport industries and for the electric insulation elements manufacturing. Similarly to epoxy and urea-formaldehyde resins, the main limitations in their application is related to toxicity and environmental issues, such as unreacted formaldehyde emission during use [249], and the lack of effective end-of-life biodegradation agents [250]. These issues have mainly been addressed with the partial substitution of the components by biopolymers [251,252]. 

The association of keratin with phenolic resins has also been studied, where feathers were the keratin source of choice. Intending to tackle the issue of preparing more sustainable fibreboards, Winandy et al. prepared medium density fibreboard (MDF) panels with aspen fibre and 0–95 wt.-% chicken feather keratin, using 5% phenol formaldehyde resin. They observed that the addition of keratin decreased the strength and the stiffness of composites compared with that of all-wood control panels. However, keratin improved the resistance to water-soak absorption, also providing fungal decay protection, probably due to hydrophobicity increase [253]. Similarly, Jiang et al. used chicken feather keratin for partially replacing the phenol (about 33 wt. %) in the phenol-formaldehyde resin synthesis, to prepare wood adhesives. Prior to the reaction, they applied two different feather protein hydrolysis methods (with and without presence of phenol during hydrolysis), two formaldehyde/phenol molar ratios (1.8 and 2.0), and three pH levels (9.5, 10.5, and 11.5). They reported that the resin formulated with keratin hydrolyzed in the presence of phenol, using a formaldehyde/phenol ratio of 2.0, and at a pH of 10.5 was the best formulation and performed as well as the neat phenol-formaldehyde resin [254].

On the other hand, Kawahara et al. utilized feathers for the production of activated carbon (AC), applying a water-soluble resol-type phenolic resin for hybridizing, preventing feather liquefaction and controlling the degree of graphitization of charcoal. The hybridization could effectively increase the yield of charcoal by over 30% and maintained the graphitization degree at approximately 0.1, suitable for the production of AC. They reported the production of materials with a surface area and iodine-adsorption capacity of 706 m^2^/g and 550 mg/g, respectively, almost twice as high in resin-free carbonized feather materials [255]. Kawahara also used similar systems to produce well-defined precursor fibres with nanoscale diameter for carbon nanofibers using electrospinning with resol-phenol formaldehyde resin, keratin and PVOH dissolved in water as the spinning dope. The author suggested that the obtained electrospun fibres could be directly carbonized to produce non-woven carbon nanofiber fabrics [256].

### 7.3. Keratin Associations with Natural Polymers and Fibres

**Figure 15:**
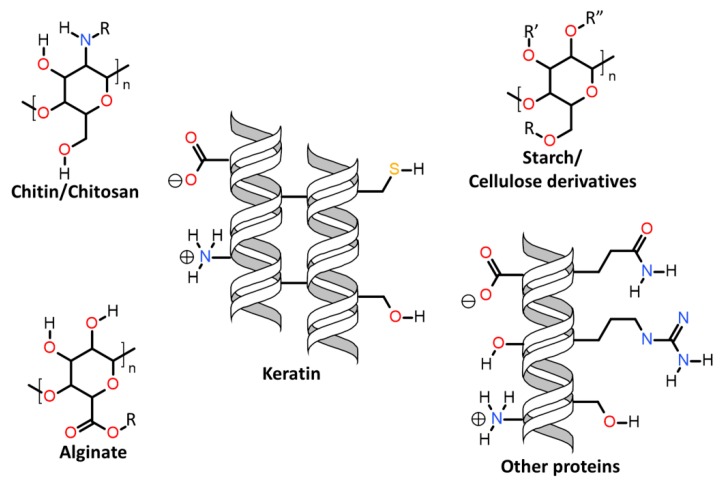
Schematic representation of the natural polymers discussed (carbohydrates and proteins), evidencing their generalized structures and available functionalities for interacting with keratin.

The use of natural polymers, especially the (partially) biodegradable ones, in polymers blends has been growing in importance, especially, but not exclusively, led by the plastic-associated environmental crisis (see Sustainability and safety assessment section). Although keratin partially fulfils this role as a biopolymer, as previously mentioned, its processability and post-processing mechanical properties are limited. Its blending with other natural polymers may lead to improved rheological properties without losing the desired biodegradability/biocompatibility. Herein are discussed the different classes of natural polymers used in association with keratin, segregating the available literature by reference polymer, and their structures and different functionalities available are represented in Figure 15.

#### 7.3.1. Keratin Associations with Carbohydrates

Carbohydrates are the most abundant natural polymers and the major structural and energy storage component in both plants and animals. They are made of long chains of glycoside-bonded saccharides (polysaccharides), which can be easily chemically or biochemically modified. For this reason, they serve as viable renewable resources for processing and manufacturing functional materials, from tissue regeneration and targeting drugs to improving food safety and packaging biodegradability [257]. At the end of this section, Table 3 summarizes the main processes used and the thermomechanical properties achieved with the keratin/carbohydrate polymer blends.

##### 7.3.1.1. Cellulose

Lignocellulosic matter is the basic building block of plants and trees, thus making cellulose a renewable and sustainable polysaccharide-based polymer of nearly unlimited supply. Cellulosic fibres have been used by mankind for thousands of years as lumber, textile and cordage, and the current industrial uses expanded exponentially ranging from paper and textiles to explosives and dietary fibres [258]. Cellulose is a linear chain of ringed glucose molecules with the repeat unit comprised of two anhydroglucose units linked together through an oxygen covalent bond, namely the 1–4 glucosidic bond. Moreover, van der Waals and intermolecular hydrogen bonds promote parallel stacking of multiple cellulose chains forming elementary fibrils that are further aggregated into larger microfibrils (5–50 nm in diameter and several microns in length), which are the main reinforcement in, e.g., trees and plants. The cellulose fibrils present regions of highly ordered (crystalline) structure and other disordered (amorphous-like) regions, and the variations of them among different cellulose sources make it possible to obtain a large variety of cellulose-derived materials [259].

When associated with keratin without any modification, cellulose/lignocellulose fibres generally produce a week interphase bonding. This was demonstrated by Barone, which prepared a plasticized keratin matrix from the reactive processing of poultry feather, glycerol, water, and sodium sulphite, and formed composites with lignocellulose fibres of varying source (corn stalk, wheat straw, banana, coffee bean husk, hemp, flax and kenaf), length (53, 246, and 589 µm), and mass fraction (0–40 wt. %). The author observed that a positive reinforcement only occurred for modulus but not stress at break, indicating resistance only under small deformations, while at large deformations, fibre pull-out was observed as a probable consequence of weak fibre–polymer interactions. High-fibre loadings reinforced the composites via lignocellulose fibre network formation, also increasing thermal stability. Consequently, the best reinforcements were observed for the application of long lignocellulosic fibres and/or high fibre loadings [260]. On the other hand, Yang et al. prepared biocomposite films, via solution casting, based on chicken feather keratin reinforced by cellulose nanocrystals (CNC), with and without dialdehyde functionalization, which reinforced, cross-linked, improved the interfacial interactions and formed a percolating nanofiller network into the keratin matrix. They reported that both aldehyde-modified and neat cellulose nanocrystals reinforced the composite films, gradually increasing the tensile strength and Young’s modulus with the increase of nanofiller content. The highest Young’s modulus and tensile strength observed were 451 and 26.2 MPa, respectively, achieved by incorporating 5 wt. % of aldehyde-modified filler, which also showed significantly increased elongation values as well as possessing the highest elongation at break (30 %) [261]. In addition, Kaur, Arshad and Ullah prepared chicken feather keratin/CNC nanocomposites with different CNC loadings (0–10%) and compared them with the application of keratin/montmorillonite. They initially solubilized the keratin in urea, EDTA and sodium sulfite solution, and further added montmorillonite or cellulose nanocrystals forming a composite that was separated by precipitation. The composite was then mixed with 20% plasticizer (1,2-butanediol for montmorillonite and glycerol for cellulose nanocrystals), 10% crosslinker (chitosan) and 3% sodium sulfite (as reducing agent) and applied for compression moulding. The authors reported that both fillers presented exfoliated to intercalated morphologies at lower contents (1 and 3wt. %), while aggregates were formed at higher concentrations (10 wt. %). Montmorillonite enhanced the tensile strength (6.7 MPa at 5% filler content), while cellulose nanocrystals improved the elongation (27.6% at 5% filler content). Crystalline melting temperatures were altered with improved thermal stabilities at low nanoparticle contents (1 and 3 wt. %) and thermal degradation of the keratin matrix was slower when montmorillonite was applied [262].

Other authors opted for cellulose modification prior to mixing with keratin, such as the case of Liebeck et al. that produced aqueous goose feather keratin hydrolysate solutions from feathers using superheated water as solvent, acquiring a high solute content in the solution (76 wt. %, at 220 °C for 120 min), and using it for producing composite membrane films with methyl cellulose as supporting material. Films with methyl cellulose/keratin hydrolysate ratios between 300/83.75 and 300/418.75 were homogeneous, with hydrolysed keratin incorporation into the semi-crystalline methyl cellulose structure, and the original keratin ordered structure was no longer present. By increasing the keratin content in the films from 1 to 5 wt. % the Young’s modulus (from 0.54 to 0.15 GPa) and yield strength (from 27.8 to 10.7 MPa) decrease, while the ultimate elongation at break (from 45.2% to 93.5%) increases, indicating a plasticizing effect of the low molecular weight keratin oligopeptides. The thermal stability of all hybrid films is higher than for pure keratin hydrolysates (~285 °C) but lower compared to neat methyl cellulose films (~359 °C) [263]. More recently, Zhou et al. prepared a polyelectrolyte complex, for encapsulating pesticides, with chicken feather keratin and carboxymethyl cellulose, via electrostatic interactions. They used avermectin as a model drug and obtained an average encapsulation efficiency of 67.06%, producing an average particle size of 386.57 nm, which presented an increased stability under UV light irradiation and about a 5-fold increase in the drug half-life. Moreover, the authors reported that the drug release mechanism was pH-dependent and was consistent with the Korsmeyer–Peppas model, and no significant toxicity difference was detected between free and encapsulated drug [264].

Exploiting the good thermal stability of chicken feather keratin, Wang, Changqin and Chen prepared a keratin-based phosphorus nitrogen-containing flame retardant by reacting keratin with melamine, sodium pyrophosphate, and glyoxal, which was tested also in combination with borax and boric acid for coating cotton (cellulose) fabric. They reported that the coating facilitated the formation of a homogenous and compact intumescing char layer, consequently producing a good synergistic effect improving the thermal stability and flame retardancy of the treated cotton fabric (40% char at 800 °C and limited oxygen index = 39.9) in comparison the bare cotton (<4% char at 800 °C and limited oxygen index = 18.0), outperforming the main commercially used flame retardants such as melamine pyrophosphate [265].

Taking advantage of the good solubility of both keratin and cellulose in ionic liquids, many authors studied these systems for blend formation for different applications, such as the case of Byrne et al., who published a series of studies using allyl-functionalized ionic liquids to prepare regenerated composites with different keratin associations with other natural polymers. They initially reported the fabrication of regenerated films of three natural polymers—raw cotton, silk and wool keratin—using ionic liquid as a recyclable solvent at 105 °C. The biocomposite films were prepared by co-solvent coagulation, where methanol enhanced the formation of keratin β-sheet structures consequently reducing the strain at break, while water produced the films with the highest stress (30.42 MPa), strain at break (1.56%) and Young’s modulus (1.76 GPa) among the composites and higher thermal degradation temperature (280 °C) than the raw cotton and the native silk and wool fibres. The improvements were attributed to the increase in intra-molecular hydrogen bonds for the biofilms [266]. Then, they also prepared regenerated cotton/duck feather composite films, which also showed enhanced stress (47.16 MPa), strain at break (11.63%) and Young’s modulus (1.66 GPa), as well as thermal stability (*T*_d_ = 284 °C) at an optimum 10 wt. % feather content. However, in this case, the authors attribute the elastic properties improvement to a larger amount of α-helix keratin in the composite films [267]. Later on, they used the same cellulose/duck feather composite to be wet-spun (extruded), from an ionic liquid solution, into fibres. Similarly to the previous systems, they obtained composite fibres, with an optimum keratin amount of 10 wt. %, presenting enhanced stress (83.07 MPa), strain at break (5.14 %) and Young’s modulus (8.73 GPa), which, applying a stretch during spinning, produced further improved stress (131.57 MPa), strain at break (6.61%) and Young’s modulus (15.56 GPa) at 13.33% stretch [268]. Similarly, Orelma et al. prepared cellulose/chicken feather keratin filaments by wet-spinning from an ionic liquid solution. Both keratin and cellulose were dissolved in 1-ethyl-3-methylimidazolium acetate and spun into ethanol to regenerate cellulose and keratin, also removing the ionic liquid. The authors observed that a small keratin addition into the cellulosic filaments improved the mechanical properties, with the highest values obtained for a cellulose:keratin ratio of 90:10 (tensile strength = 142.4 MPa, Young’s modulus = 7.5 GPa and elongation = 19.3%), whereas high keratin additions resulted in a reduced mechanical performance. The morphology of cellulose changed from cellulose I to II and the β-sheets in feather keratin unfolded to an amorphous structure upon dissolution and regeneration. They also observed that feather keratin was only partially coagulated with ethanol, and consequently, not all the keratin content applied was incorporated into the filament [269]. In addition, using the same ionic liquid dissolution approach, Tran and Mututuvari prepared composites with cellulose and sheep wool keratin, comparing also with chitosan and keratin, by dissolution in 1-butyl-3-methylimidazolium chloride ionic liquid, followed by casting and washing in water for ionic liquid removal. They produced a recyclable dissolution process that preserves the polymers’ original chemical structure. However, keratin increased the amount of β-sheet conformation and the α-helixes were disrupted by dissolution in ionic liquid. They observed that the best tensile strength (~38 MPa) and thermal stability (*T*_d_ = ~305 °C) were presented by the cellulose/keratin composites at 60–75 wt. % cellulose loading [270]. They also used the same systems to encapsulate and release drugs such as ciprofloxacin (CPX), and found out that the drug release rates are decreased proportionally to the increase in the keratin amount, allowing controlled drug release. They also observed that the mechanical strength was improved by adding cellulose, while hemostasis and bactericide properties were developed with the addition of chitosan [271].

##### 7.3.1.2. Chitosan

Chitosan is a linear polymer occurring naturally only in certain fungi (Mucoraceae), presenting a structure similar to cellulose but chemically composed of glucosamine and *N*-acetylglucosamine monomers linked through β-(1−4)glycosidic linkage [272]. Chitosan is most commonly obtained by the deacetylation (under alkaline conditions or enzymatic hydrolysis) and depolymerization of chitin. Chitin/chitosan is the second most abundant form of polymerized carbon in nature (after cellulose), since it is present in the exoskeleton and internal structure of invertebrates such as molluscs, crustaceans, insects, fungus and algae. Since it is a cheap and abundant source of renewable, biocompatible and biodegradable polymer, it has been intensively investigated for applications, e.g., as chelating agent, water treatment additive, drug carrier, and wound-healing agent, also in biodegradable adhesive tapes and in membranes [273].

Differently from the case of cellulose/keratin associations, chitosan strongly interacts with keratin; however, for improving the elongation properties, often, these blends demand plasticization. Tanabe et al. demonstrated such a feature when developing wool keratin/chitosan composite films via solution casting (in 75% acetic acid), and observed that the addition of 10–30 wt.-% chitosan produced strong and flexible films (tensile strength 27–34 MPa, elongation 4–9%). They also observed that the application of glycerol (20 wt. %) to the composites decreased the tensile strength (from 14 to 9 MPa) but considerably increased the elongation (from 18% to 31%). Moreover, the swelling behaviour and mechanical properties after swelling were improved in the composite film, also producing antibacterial properties and promoting mouse fibroblast cells attachment and proliferation [93].

Martínez-Hernández et al. reinforced a sorbitol (1% vol.) plasticized starch/chitosan (95/5 wt. %) matrix with three different kinds of keratin derived from chicken feathers: (i) long fibres obtained according to a patented process [274], and (ii) short fibres and (iii) ground rachis prepared by cutting manually barbs and barbules from quill and finely grinding in a hammer mill. These were added separately at 5, 10, 15 and 20 wt. % to the starch/chitosan matrix and the composites were processed via solvent casting. They reported that the addition of keratin enhanced both the thermal stability and thermomechanical properties of the matrix, where the optimum keratin amount was 5 wt.-%, and the contributions of the different fillers were in the following descending order (storage modulus at 5 wt. % keratin): short fibres (1142 MPa) > long fibres (582 MPa) > rachis (527 MPa) [275]. Later on, the same group used potato starch, chitosan and chicken feather keratin for processing biodegradable films via solvent casting followed by melt extrusion. They initially prepared two different solutions: (i) starch aqueous solution (3.8%) plasticized with sorbitol (1 vol. %) and (ii) chitosan acetic acid solution (0.2%), which were mixed together with the addition of 5–10 wt. % of feather keratin (with or without NaOH treatment), followed by solvent casting, drying and milling the resulting films for extrusion. They obtained films with excellent keratin dispersion, with decreased water solubility by increasing the keratin ratio. Composites without feather (NaOH) treatment presented significant increases in storage (up to 137%), elastic modulus (up to 8160%) and maximum strength (up to 3800%), while composites with feather treatment presented increases up to 192% in storage modulus, 7250% in elastic modulus and 3150% in maximum strength. However, the increment of keratin in the composites decreased the degradability rate [276].

Chitosan was also often used to improve or repair the properties of woollen fabrics via interactions with the wool keratin. One approach used was crosslinking via esterification and transamidation reactions using pad–dry–cure treatment of woollen fabrics with potassium permanganate oxidation followed by crosslinking with acetic acid, as demonstrated by Hsieh et al. They observed beneficial effects on the antimicrobial and antiseptic properties but detrimental effects to the fabric softness, yellowness, stretching resistance and elongation [277]. Moreover, Ghosh, Grosvenor and Dyer used 2-hydroxyethyl cellulose (neutral), chitosan (cationic) and alginate (anionic) as repair agents to improve the mechanical properties and the morphology of woollen fabrics after alkaline damage. They reported that chitosan was the most effective polymer for remedying the mechanical strength, fibre integrity and surface wettability after severe alkali damage, most likely by the formation of strong ionic/covalent interactions between the keratin negatively charged sites (caused by lipid removal) and chitosan [278]. Ranjbar-Mohammadi, Bahrami and Arami acylated woollen fabric with succinic anhydride and grafted it with chitosan (15 g/L) under sonication (120 W, 20 kHz for 60 min). The chitosan grafted-acylated wool fabrics presented a better dyeing ability, lower shrinkage and antibacterial properties against Gram-positive (Staphylococcus aureus) and Gram-negative (Pseudomonas aeruginosa) bacteria [279]. 

In contrast, Shanmugasundaram et al. used this strategy for dip-coating a spun lace nonwoven fabric (polyester/viscose blend 30/70 wt. %) using three different chicken feather keratin-based biopolymer solutions for producing wound dressings: (i) neat keratin, (ii) keratin-sodium alginate (90/10 wt.-%) and keratin-chitosan (90/10 wt. %). The authors reported that both keratin association presented positive antibacterial effect against Gram positive Staphylococcus aureus and Gram negative Klebsiella pneumoniae and Escherichia coli with a large inhibition zone. When applied to an in vivo model (Albino Wistar rat), they also exhibited good support for cell viability and a strong cytocompatibility, promoting a complete wound in the following order: keratin/chitosan (15 days) < keratin/sodium alginate (17 days) < keratin (21 days) < control (23 days) [280].

Similar crosslinking strategies, applied on woollen fabrics, were used to associate chitosan with chicken feather keratin. As demonstrated by Selvamurugan et al., chicken feather-based keratin (nano)particles could be prepared via glutaraldehyde crosslinking and added to a chitosan matrix, forming chitosan/keratin scaffolds (by freeze-drying process) presenting porous architectures. They observed that despite the good chitosan/keratin interaction, the semi-crystalline structure of chitosan was not altered. However, the biodegradation and protein adsorption were significantly increased by adding keratin to the scaffolds [281]. In addition, Ma et al. applied chicken feather keratin (micro)particles, extracted using L-cysteine and ball milled after freeze drying, into chitosan to form composite membranes. They observed that the incorporation of keratin into chitosan decreased the contact angle from 98.18 to 58.28, while the tensile strength (6568 MPa) and elongation at break (15%) reach maximums at 6% keratin content, representing increases of 80% and 88%, respectively, when compared with the pristine chitosan membrane [282]. Similarly, Simchi et al. prepared an injectable nanocomposite hydrogel by conjugating a copolymer (Pluronic F127, PEO_99_–PPO_65_–PEO_99_) with chitosan and crosslinking with merino wool keratin. They used genepin as a crosslinker between chitosan and keratin to obtain porous (D = 5–100 µm) hydrogels with tuneable viscoelasticity and good cell viability (>90%). They also applied LAPONITE^®^ nanoparticles as reinforcing agents, which affected the hydrogel porosity and improved the elastic modulus and biostability up to 6-fold [283]. The same group further applied an adaptation of this system to produce core-shell electrospun fibres containing Aloe Vera extract, using the co-axial electrospinning technique. The core was composed of PEO/herb (D ~209 nm) and the shell consisted of PCL/chitosan/keratin (thickness ~91 nm). The authors observed that the co-axial electrospun core-shell structure improved tensile strength (~5 MPa) and elongation, where keratin positively contributed to the tensile strength, but reduced the elongation [284].

Only one example could be found on the association between chitosan and human hair (α-)keratin, where Lou et al. combined chitosan-azide (50–75 wt. %) and human hair keratin (25–50 wt. %) using UV irradiation crosslinking, producing reticulated composite membranes. Especially at high keratin content (50 wt. %), they reported an increase of fluid (PBS buffer) absorption (>10 wt. %), good tensile strength (26.33 MPa), an increase in cell adhesion and proliferation, and good biodegradability and biocompatibility when implanted subcutaneously in mice [285]. Similarly, only three examples of an association between chitosan and keratin from bovine horns or hooves could be found. Madhan et al. prepared scaffolds via a freeze-drying process made of bovine hooves keratin (25%), chitosan (25%) and gelatine (50%), presenting high thermal denaturation temperatures (200–250 °C), tensile (96 kPa) and compression strength (8.5 kPa) similar to the reference collagen scaffolds [286]. Sehgal et al. observed that at a ratio of 66.6% horn keratin to 33.3% chitosan, the blend presented a tensile strength of 1.58 MPa and 21.63% elongation at break [287]. On the other hand, Sivagnanam et al., also using horn keratin, observed that the use of larger amounts of chitosan, from 50% to 75%, increased the tensile strength (from 7.40 to 21.14 MPa) and the tensile modulus (from 0.46 to 3.15 MPa), while decreasing the elongation at break (from 16.03% to 6.19%). They also observed the drug release effect of the blend, where in vitro release of the topical antibiotic Mupirocin indicated a burst release of 32% within the first hour, followed by sustained release at the end of 92 h (64%) [288].

##### 7.3.1.3. Alginate

Alginate is an anionic linear block copolymer containing (1,4)-linked β-D-mannuronate and α-L-guluronate residues, naturally produced by brown seaweed. Alginates extracted from different sources differ in block composition and length, offering more than 200 different alginates that are currently being manufactured. Due to its biocompatibility, relatively low-cost and gelation properties, alginate has been especially attractive for medical and pharmaceutical applications, such as tissue engineering, drug delivery and antioxidant/antimicrobial pharmaceutical packaging [289]. 

Only four studies directly associating alginate with keratin to prepare composites or blends were found, with the exception of previously discussed studies in which alginate was not the main contributor for system improvement [278,280], or was a secondary actor for producing keratin/alginate solutions for fibre spinning [290]. 

In one of the studies, Tanabe et al. used the weak compatibility between keratin and calcium alginate, via a combined particulate-leaching and the freeze-drying method, to prepare highly porous (98.9% porosity) wool keratin (~55%)/calcium alginate (~45%) sponges. The porosity was formed by alginate leaching, leaving a keratin sponge that supported the attachment and the proliferation of mouse fibroblast cells [291]. 

Gupta and Nayak prepared polymer blend films where sodium alginate was the continuous phase (up to 90 wt. %) with the addition of 10 wt.-% of chicken feather keratin plasticized with 2 wt. % of glycerol, by solution casting technique. They found out that this alginate/keratin ratio produces the film with the highest tensile strength (0.38 MPa) and elongation (59.5%) [292]. On the other hand, in another study, Yin et al. applied chicken feather keratin as the continuous phase (50–90 wt. %) with sodium alginate (10–50 wt. %), plasticized with 24 wt. % sorbitol, presenting good interaction between the polymers, mainly via H-bonding and homogeneous structure. The addition of sodium alginate to keratin proportionally enhanced the tensile strength (up to 16.30 MPa at 50 wt. % sodium alginate) and maintained the extensibility of the films (9.41% at 50 wt. % sodium alginate). However, the water vapour permeability reached its lowest level at the 30 wt.-% sodium alginate ratio [293].

Finally, Srihanam et al. used the water-in-oil (W/O) emulsification-diffusion method for preparing keratin, alginate and keratin/alginate blend microparticles, where the blend solutions were the water phase and ethyl acetate was the oil phase. They obtained an optimum keratin solution concentration of 1.6%, which was blended with the same concentration of the alginate solution for the microparticles construction. The microparticles presented different shapes, from spherical to bowl-like and from porous to hollow, with varying sizes with the blend ratios. They reported that the interaction between functional groups of keratin (amino groups) and alginate (hydroxyl groups) was the main factor for both β-sheet structure and *T*_d_ values of the microparticles, with both properties increasing with increasing the alginate content [294]. 

##### 7.3.1.4. Starch

Starch is naturally produced in plant seeds, rhizomes, roots and tubers in the form of semi-crystalline granules with unique properties for each plant, but with the same two polyglucans as basic components, namely amylose and amylopectin. The molecular structures of amylose and amylopectin consist of glucose residues connected through α-(1,4)-linkages to long chains with a α-(1,6)-branches. While amylose presents longer chains and fewer α-(1,6)-branches, amylopectin has shorter chains and many α-(1,6)-branches, resulting in a complex three-dimensional structure [295].

Thermoplastic starch or plasticized starch is the processed starch from varied sources, e.g., corn, wheat, rice and potatoes, via extrusion and injection units. However, it presents many limitations for products development due to its poor mechanical properties (brittle) and high sensitivity to moisture (rapidly degradable). On the other hand, these process flaws can be advantageous when starchy materials are used as additives in blends with other polymers, increasing their biodegradability and decreasing the production cost [296]. 

Excluding two other studies discussed previously, associating another natural polymer (chitosan) [274,275], only the study of Schartel et al. discussed the direct association between keratin and starch. They used animal hair keratin fibres derived from tannery waste as fillers to prepare biocomposites with a commercially available thermoplastic starch-polyester blend, where also ammonium polyphosphate (APP) was added as a flame retardant. Keratin alone acted as a good flame retardant and had the effectiveness improved with the addition of APP. With respect to the mechanical properties, the addition of keratin to thermoplastic starch produced materials with increased Young’s modulus (229 MPa) but decreased tensile strength (10 MPa), strain at break (26%), tenacity (2 MPa) and Izod impact resistance (81 J/m). However, these values improved with the addition of 10 wt. % of APP (247 Mpa, 12 MPa, 54%, 6 MPa and 118 J/m, respectively, for tensile strength, strain at break, tenacity and Izod impact resistance) [297].

#### 7.3.2. Keratin Association with Other Proteins

Proteins are complex amino acid-based structures already long-known for naturally occurring in all six kingdoms of life (plants, animals, protists, fungi, archaebacteria and eubacteria). There are twenty-two different proteinogenic amino acids, i.e., that can join together via peptide bonds and build chains known as proteins [298]. Proteins display critical structural and bioactive properties in plants and animals that were adapted to specific uses for millions of years. Different proteins were developed to have different useful functions, such as varying cell compatibility and mechanical properties. For this reason, natural in vivo associations between different proteins are often found, e.g., collagen and elastin associations providing combined strength and toughness for specific body tissue functions [299]. Thus, it is fair to say that proteins were naturally developed/adapted for blending with other materials, and this property can be used in benefit of materials engineering for improved processability and final materials properties. Herein, the literature concerning the associations between keratin and other proteins is discussed, categorizing them by the proteins that keratin was associated to. At the end of this section, Table 4 summarizes the main processes used and the thermomechanical properties achieved with the keratin/protein polymer blends.

##### 7.3.2.1. Collagen and Gelatine

About 29 different types of collagen have been identified to date, but all of them present the common feature of a glycine as the third residue within a X-Y-Gly amino acid sequence, where X and Y are most commonly represented by proline and hydroxyproline. The collagens type I-III, V and XI are fibrillary, and type I is the most commonly used in biomaterials development due to its natural abundance. Collagen fibrils are difficult to extract and isolate, however, their hydrolytic breakdown produces three polypeptide strands, known as gelatine. It presents an amphiphilic characteristic due to alkaline and acidic amino acid residues forming thermally reversible networks in water and having been demonstrated to promote tissue regeneration [82]. Due to these characteristics and their superior cytocompatibility when compared to keratin [196], collagen and gelatine have been used mainly for fibre coating and preparing scaffolds for biomedical applications, where hair and wool (α-)keratin were the sources of choice. 

Using microbial transglutaminase (TGas)-mediated crosslinking of gelatine on the KMnO_4_-pretreated surface of wool, Fan et al. produced smoother fibres with reduced area shrinkage (1.92 ± 0.15%), increased tensile strength (335 N or ~26.8 MPa) and anti-felting ability that improved the washing durability [300]. 

Using the solvent-casting technique, Prasong and Wasan prepared human hair keratin/gelatine blend films and observed improved thermal properties in comparison to neat keratin [301]. Similarly, Thonpho and Srihanam blended extracted human hair keratin with collagen, gelatine, sericin and starch, individually, and obtained films without phase separation, with the exception of the keratin/starch blend. They observed that at low keratin ratios, its structure changed from β-sheet to random coil, also decreasing the thermal stability. They also observed that the blends’ behaviour for drug release (chlorhexidine) was independent of the structural changes, with the drug release rate increasing in the following order: keratin < keratin/sericin < keratin/starch < keratin/collagen < keratin/gelatine [302]. The association between gelatine/keratin (hair or wool) (90/10 wt. %) and gelatine/*Bombyx mori* silk (88/12 wt. %) also made it possible to obtain hemocompatible three-dimensional scaffolds with highly interconnected pores, via the freeze-drying technique, as described by Arul et al. The gelatine/keratin composite presented higher porosity (366 ± 49 μm), a better mechanical strength (about 0.23 MPa) and sustained a more controlled drug release (sodium diclofenac), in comparison to the gelatine/silk composite [303].

##### 7.3.2.2. Soy and Wheat Protein

Soybean protein is a globular protein composed of two main subunits (conglycinin 7S and glycinin 11S) that contain regions of non-polar amino acids (e.g., alanine, valine, and leucine), basic amino acids (e.g., lysine and arginine), and non-charged polar residues (e.g., cysteine and glycine). Presenting a globular structure gives soybean protein stability and resistance to hydrolysis, making it especially interesting for biomaterials engineering [82]. Due to its biodegradability, abundant renewable sources and available functional groups that produce adhesive properties [304], it has been successfully applied for surface modifications, polymer blending [305] and fibres fabrication [82,306]. 

Wheat protein, also known as wheat gluten, is composed of different protein fractions with low *M*_w_ (albumins and globulins) and high *M*_w_ (glutenins and gliadins). Glutenin is one of the largest naturally occurring polymers (*M*_w_ > 10^7^), also presenting predominant intermolecular disulfide bonds, and is the main reason why gluten provides improved viscoelastic properties to bread dough [307,308]. Moreover, wheat gluten is a by-product of starch fabrication [307], thus representing an abundant and inexpensive source of biopolymer for applications like films, gels, foams and bioplastics [309].

Enzymatic hydrolysis (Alcalase with pH 8.2 and 55 °C for 60 min) and cationization (with epoxypropyldodecyl dimethyl ammonium chloride) to wheat gluten has been used for structural recovering damaged human hair by Zhao et al. The authors applied the cationization to increase the isoelectric point of the gluten hydrolysate from 7.0 to 10.0, facilitating the adherence to the surface of hair at pH 5–6 (ideal for hair care products). The quaternized gluten hydrolysate presented excellent properties in recovering damaged hair, making the surface of hair smooth and compact [310].

On the other hand, Guerrero et al. blended hydrolysed chicken feather keratin (3, 6 or 9 wt. %) with soy protein (91, 94 or 97 wt. %), plasticized with glycerol (30 wt. %), to prepare transparent films via casting and compression moulding. They observed that the incorporation of hydrolysed keratin proportionally improved the thermal stability of the films obtained with both processing methods, but the compression moulding favoured tensile strength enhancement (from 7.47 to 9.52 MPa with 9 wt. % keratin) and elongation decrease (from 131% to 94% with 9 wt. % keratin). The water uptake of the keratin containing films remained constant after 24 h, indicating a high stability and structural integrity of the manufactured films [311].

##### 7.3.2.3. Silk Fibroin

Silk consists of two main proteins that are naturally produced by worms, insects and arachnids: i) silk sericin, an adhesive protein located on the outside of silk strands that makes up to 35% of silk cocoons; and ii) silk fibroin, a protein predominately composed of hydrophobic units (glycine, alanine, and serine) forming β-sheets that infer a high tensile strength, and hydrophilic blocks consisting of charged amino acids that give its deformability [312,313]. Silk fibroin obtained from the Bombyx mori silkworm is the most commonly used type, due to low cost and availability, presenting excellent biocompatibility, biodegradability, mechanical stability, and oxygen and water vapour permeability, thus often used as scaffolds in tissue regeneration and wound healing [82].

The association between wool keratin and *Bombyx mori* silk fibroin to produce blend films via solution casting in aqueous and formic solutions has been demonstrated by Vasconcelos, Freddi and Cavaco Paulo. They observed that after casting the aqueous solutions of the neat proteins, the keratin was mainly constituted of α-helix and random coil conformations while the fibroin was prevalently amorphous (random coil conformation). On the other hand, both keratin and fibroin casting from formic acid solutions had an increased amount of β-sheets. However, fibroin/keratin blends did not follow the additive rules due to the intermolecular interactions formed. When the blends were exposed to in vitro enzymatic incubation with trypsin, the blend film cast from water solution underwent a slower biological degradation than the films cast from formic acid, and the degradation was increased for larger keratin fractions. The films obtained from formic acid solutions presented the best mechanical properties. However, they decreased with an increase in the keratin content, with the tensile modulus varying from 2.89 GPa for the neat silk to 2.01 GPa for 50 wt. % addition of keratin [94].

Using the physical crosslinking between Bombyx mori silk cocoon fibroin and sheep wool keratin, Hu et al. prepared biocompatible hydrogels via two approaches; (i) by ultrasound-induced gelation of the proteins’ aqueous solution, and (ii) by naturally assembled silk/keratin mixtures through long-term rest of the aqueous solution. The silk/wool blend solutions formed perfectly interconnected hydrogels when the wool content was below 30 wt. % for the sonication approach and below 10 wt. % for the natural gelation, and for both approaches the β-sheet crystallinity increased with increasing the silk content. They also observed that ultrasound application can significantly enhance the crosslinker formation and avoid silk/keratin phase separation [314].

Using the principle of heat generating by moisture absorption and the secondary structural synergy between wool keratin and silk fibroin, Youbo et al. developed a self-heating textile fibre using wool keratin, protein/viscose fibre (PVF) and cotton pulp as raw materials with silk protein used as crosslinker between keratin and cellulose acetate. The PVFs were prepared by wet spinning, presenting improved properties proportional to the protein addition, where they kept an optimum protein content of about 2.5%. In addition to the self-heating property, the PVF presented the requirements for textile fibres, such as high breaking strength (2.44 cN/dtex), high elongation-at-break (27.5%), high moisture regain (12.6 8%) and high hand feel score (8.4) [315].

##### 7.3.2.4. Associations between Different Keratin Sources

As previously discussed in this review, the differences in peptides compositions and molecular weights between keratins obtained from different sources, affecting strongly their crystalline structure, can be comparable to completely different polymer systems. In fact, in vivo, taking dermal tissue keratin as an example, the polypeptides’ composition heterogeneity can be observed between species, within a species, and in different areas of the skin of a single member of a species [316]. Thus, blends of different keratins can neither be referred to as a homologous polymer blend due to the peptides heterogeneity, nor as an isomorphic polymer blend due to the heterogeneous crystalline structure, but simply as a polymer blend [317].

Unfortunately, this is a very poorly explored topic, which is open for exploitation in natural polymer systems properties tuning. However, one example of this approach was demonstrated by Wang and Peng, who treated wool fibres with chicken feather keratin, plasma, and their combination. The authors observed improvements in the antifelting performance, wettability, and dyeability of the wool when modified by the combination of keratin with plasma, caused by the modification of the wool surface chemical composition and morphology/structures with the plasma pre-treatment, especially by the increase of thiol and hydroxyl groups available. Consequently, the plasma pre-treatment followed by keratin coating presented the best anti-felting performance, wetting properties, and dyeing behaviour [318].

## 8. Summary and Outlook

Keratin has been used for centuries both as a biomedical and structural material, however, a modern scientific comprehension of the latter is rather recent, especially as a consequence of keratin’s difficult processability. Due to its abundance as an industrial side stream, it could give an essential impulse for the engineering of bio-based materials and serve as replacement or improvement of commodity synthetic polymers. The improvements of keratin extraction processes made a great contribution to increasing its application range in a sustainable way, especially concerning ionic liquid-based extractions or steam explosion methods that do not depend on the use of harmful solvents.

Within this context, keratins from different sources have been demonstrated as structurally different (due to molecular weight, crystallinity and conformation differences), in such an extent that they need to be treated as different matrices/fillers when applied to blend systems. This, in fact, makes the keratin exploitation even more interesting since once these distinctions are made, this allows a myriad of reinforcing/enhancing effects. Among the most important property-altering keratins’ differences are: the amount of sulfur-containing segments, such as cysteine, allowing covalent crosslinking within a polymer blend; and the ratio between the long/flexible (α-) and short/stiff (β-) keratin, producing different ratios of stiffness/toughness reinforcement. The variation of the abovementioned ratios also caused the formation of a complex interphase construction, involving varying amounts of covalent bonds, ionic bonds, H-bonds and physical interactions.

Blending keratin with other polymers has shown promising results within most polymer classes, tackling at least partially both the processability issues of keratin and the environmental issues of synthetic polymers, and creating access to cost-effective biopolymer-based blends.

Considering blends with synthetic thermoplastic polymers, keratin has found a vast field of applications in fibre casting and fibre coating, both for advanced clothing textiles and in biomedical applications (membranes, scaffolds and hydrogels). Keratin has been shown to improve processability in different methods, also allowing to control the water adsorption, the fibre crystalline structure and the mechanical properties, where α-keratin improves fibre stretchability and β-keratin produces stiffer fibres. Keratin has been vastly applied for fibre coating in association with polyacrylates, PAN and PAM, and for fibre casting with PEG, PEO, PVOH, PA6, PCL and PLA. The preparation of blend films of keratin using solvent casting, melt-mixing and/or compression moulding has also yielded promising results, where keratin has shown to produce stable films with PVOH, PA6, PLA, PEO, PHBV and TPU. The association between hair α-keratin and TPU has been shown to be especially effective, yielding a promising candidate for artificial skin via melt-mixing and compression moulding [209]. However, only feather (β-) keratin has yielded good reinforcing effects, both as a filler or matrix, in polymers with already good thermomechanical performances (e.g., PE, PP, PVC and PLA). The preparation of these blends has been shown to be compatible with standard scalable methods, such as melt-mixing, forming blends with reduced density and flammability, and increased thermal stability, elastic modulus and yield stress. This could allow the application of keratin/thermoplastic polymer blends both in already-established processes and emerging techniques such as additive manufacturing. It is also worth mentioning that using the thiol moiety, keratin could also serve as a crosslinker in different graft-copolymerization processes, and feather keratin acted as support and initiator for olefin polymerization without the need for any other initiator or catalyst [111]. Finally, keratin inflicted a general cytocompatibility improvement to the synthetic polymers it was associated with, potentializing their used in biomedical applications.

Concerning the effect of keratin in elastomers and thermosets, its application in association with butadiene copolymers increased the rubber crosslinking efficiency, promoting mono- and disulfide bonds, which are more stable than polysulfide bonds. The association of keratin with epoxy resins was mainly explored using feather (β) keratin as a reinforcing (filler) phase, producing blends that are ideal for sustainable printed circuit boards (very low dielectric constant), acoustic insulation (low density) and adhesives (available thiol moiety). In association with urea-formaldehyde and phenol-formaldehyde resins, keratin allowed the reduction of production costs and formaldehyde emissions for bonding particle boards and MDF, also providing resistance to water-soak absorption and fungal decay protection.

Similarly to the case of the synthetic polymers, the preparation of blends between keratin and carbohydrates yielded many different products (from films and fibres to membranes and scaffolds), in most cases improving the processability in comparison to the neat polymers. The formation of blends was highly dependent on the functional groups of the polysaccharide, where, without modification or compatibilization, cellulose, alginate and starch produced weak interphase bonding while chitosan strongly interacted with keratin. The keratin/chitosan blends should be highlighted, where improvements in thermal stability, flame retardancy and thermomechanical properties were observed, especially for keratin as the minor (filler) phase, producing good wound healing properties. The increment of large amounts of keratin was less effective at enhancing properties and also decreased the already good degradability rate of chitosan.

The association of other proteins, such as gelatine and collagen, with keratin has been mainly explored for fibre coating and preparing scaffolds for biomedical applications, where hair and wool (α-) keratin were the sources of choice. Moreover, although still poorly explored, soy and wheat protein (a by-product of starch fabrication) were demonstrated to be abundant and inexpensive sources of biopolymer for associating with keratin, yielding applications like films, gels, foams and bioplastics. Moreover, the combination of keratin with silk fibroin could not surpass the outstanding mechanical properties of the neat silk. However, it allowed the substitution of a considerable amount of fibroin by keratin without dramatic thermomechanical properties loss, prospecting the production of cheaper silk-based material via casting or crosslinking, ranging from hydrogels to self-heating textiles. Finally, concerning the association of different keratin sources, only wool fibres were demonstrated to improve antifelting properties when coated with feather keratin, prospecting interesting textile applications. However, this is a completely open topic for exploration with many opportunities to prepare natural biomaterials with tuneable properties.

## Figures and Tables

**Table 1:** Summary of thermomechanical properties and preparation/processing conditions of keratin/thermoplastic polymer systems.

Associated Polymer	Source	Amount (wt.-%)	Additives *	Mechanical Properties (MPa) **	Thermal Properties (°C) ***	Preparation Method	Obs. ****	Ref.
**PP**	feather	30	MPR/APS	*TS* ~ 35; *E* ~ 2500; *σ* ~ 50; *E_bend_* ~ 3000; *IS* ~ 55(J/m)	-	melt mixing	-	[108]
		30	MAPP	*E* = 3590; *TS* = 40.71; *E_bend_* = 3530; *σ* = 75.20	-	compression moulding	composite panels	[104]
		5–15	-	*E* = 1167–1451; *TS* = 40.71; *E_bend_* = 3530; *σ* = 75.20	*T_g(α)_* = 109–137; *T_m_* ~ 156–169; *T_d(max)_* ~ 300–400	melt mixing/compression moulding	-	[110]
	wool	30	MAPP	-	*T_d(max)_* ~ 450	melt mixing	fire retardant	[107]
**LDPE**	feather	20	-	*E* ~ 80; YS ~ 17	-	melt mixing		[106]
**PEO**	wool	20–60	-	-	*T_m_* = 64.4–53.5; *T_c_* = 41.6–9.7	solvent casting	-	[95]
		10–70	-	*E* = 12–7; *σ_break_* = 4.7–1.6	*T_m_* ~ 200–220	Electrospinning	fibres	[116]
	hair	90	EGDE		*T_d(max)_* ~ 290–420	Electrospinning	fibres	[119]
	feather	90	graphene	*E* ~ 1200; *E_r_* ~ 12000; *H_i_* ~ 570	-	solvent casting	films	[120]
**PEG**	hair	43	-	*E* ~ 0.030; *E_comp_* = 0.045	-	photo-crosslink (thiol-norbornene “click”)	hydrogel	[121]
**PMMA**	wool	-	-	*TS* ~ 152	*T_m_* ~ 230; *T_d(50%)_* ~ 390	graft-copolymerization	fibres	[139,140]
**HEMA**	wool	-	-	*L_max_* ~ 0.20(*kN*), *ε_max_* = 35.71(%)	-	graft-copolymerization	fibres	[141]
**PBzMA**	wool	-	-	*TS* = 377(g), *ε_break_* = 40.0(%)	*T_m_* ~ 232–278, *T_d(10%)_* = 300, *T_g_* ~90	graft-copolymerization	fibres	[143]
**PMMA** **PEMA;PBMA** **PHMA**	feather	-	-	*σ_break_* ~ 7.0; *ε_break_* = 45.5	*T_d_* = 380–520	graft-copolymerization/compression moulding	films	[146]
**PAM**	wool	-	-	*TS* = 247(g); *ε_break_* = 1.7(%)	*T_d(10%)_* ~ 235; *T_d(50%)_* ~ 665	graft-copolymerization	fibres	[148]
**PVOH**	wool	10–30	-	*T_n_* = 1.49–1.57(*cN/dtex*); *ε_break_* = 22.1–41.1(%)	*T_d(max)_* ~ 260	wet-spinning	fibres	[159]
		5–25	-	*TS* = 15.8(*cN*), *ε_break_* = 77.6(%)	*T_d(5%)_* ~ 230	wet-spinning	fibres	[160]
	Feather	20	Citric acid/glyoxal	*TS* ~ 12; *ε_break_* ~ 150(%)	*T_m_* ~ 60, 200 and 270; *T_d(5%)_* ~ 250; *T_d(50%)_* ~ 360	Electrospinning	fibres	[162]
		80	DAS	*TS* ~ 15–21; *ε_break_* ~ 2.5–9.0(%)	*T_g_* = 100–125; *T_m_* ~ 220; *T_d_* ~ 250	solution casting	films	[165]
		50–90	Tris	*E* ~ 16–2007; *TS* ~ 4–23(%); *ε_break_* = 2–262(%)	-	solution casting	films	[167]
	hair	92	β-mercapto ethanol/IL/thioglycolic acid	*TS* ~ 6	*T_m_* ~ 290	Electrospinning	fibres	[163]
**PA6**	feather	20–80	-	*E* = 825–1444; *S_n_* = 1.42–2.86(N m^−1^)	-	spin-coating	films	[173]
**PCL**	hair	10–40	-	*E* = 4.5–8; *σ_break_* = 1–2	-	Electrospinning	membranes	[179]
		10	MgO	*E* = 5.5; *TS* = 3.0;	-	Electrospinning	membranes	[180]
		30	Glutaraldehyde/Ca_3_(PO_4_)_2_	*E* = 25.92; *TS* = 16.53; *ε_break_* = 153(%)	-	Electrospinning	scaffolds	[181]
**PLA**	Wool/fleece	1–5	-	*σ_break_* = 13–28; *ε_break_* = 9–197(%); *E* = 420–1200	*Tg* = *61*–64; *T_c_* = 100–109; *T_m_* ~ 180; *T_d(max)_* ~ 300–360	solvent casting	films	[185]
	feather	30	MPR/APS	*TS* ~ 50; *E* ~ 4500; *σ* ~ 90; *E_bend_* ~ 9000; *IS* ~ 25(J/m)	*T_g_* ~ 55; Tc ~ 87; *T_m_* ~ 170; *T_d(50%)_* ~ 400	melt mixing	-	[108]
		2–4	chitosan	*E* ~ 2300–3300; *TS* ~ 35–65; *ε_break_* = 1.3–3.8(%); *IS* ~ 7–11(KJ/m^2^)	*T_d(50%)_* ~ 330–365	melt mixing	-	[188]
		50–60	copolymer blend/PEG400	*E* ~ 1500–2100; *TS* ~ 5–22; *ε_break_* = 1.4–1.9(%)	*T_g_* ~ 47–66; *T_m_* ~ 145–179; *T_d(50%)_* ~ 321–325	melt mixing/compression moulding	films	[189]
		5–25 (vol.-%)	-	*E* ~ 2800–3100; *TS* ~ 21–36; *ε_break_* = 1.6–1.9(%)	-	melt mixing/compression moulding	films	[190]
**TPU**	feather	11–21	PPG	-	*T_g_* ~ 50; *T_m_* ~ 133; *T_d(max)_* ~ 418–424	graft-copolymerization	membranes	[204]
		10–80	-	*E_c_* = 60–200; *TS* ~ 5–29; *ε_break_* = 183–276(%); *E_comp_* = 40–145(scaffold)	*T_g_* ~ −42	solvent casting	scaffolds	[206]
		10–70	-	*E* ~ 100–1190; *ε_max_* = 0.6–2.5(%)	*T_g_* ~ −45; *T_d(5%)_* ~ 249–309	solvent casting/compression moulding	films	[207]
	hair	0.5–10	-	*E* = 31.4; *TS* ~ 18.5; *ε_break_* = 570(%)	-	melt mixing/compression moulding	artificial skin	[209]
**TPUU**	feather	40–75	bis(4-aminophenyl) disulphide/bis(4-aminophenyl) methane	*E* ~ 67–408; *TS* ~ 2.8–11.2; *ε_break_* = 1.9–30.7(%)	*T_d(5%)_* ~ 204–237; *T_d(50%)_* ~ 340–361	melt mixing/compression moulding	films	[210]
**PHBV**	feather	0.5–50	-	*E* ~ 540–865; *ε_break_* = 2.8–4.7(%)	*T_c_* ~ 102; *T_m_* ~ 147–157	melt mixing/compression moulding	films	[197]
		0.5–5	-	*E* ~ 770–1840;*TS* ~ 16–30; *ε_break_* = 0.7–5.3(%)	*T_c_* ~ 101–115; *T_m_* ~ 136–184	electrospinning/solvent casting/melt mixing/compression moulding	films	[198]
**PBAT**	Feather	50–60	PEG400	*E* ~ 300–600; *TS* ~ 3–5; *ε_break_* = 1.8–2.8(%)	*T_g_* ~ −30; *T_m_* ~ 117; *T_d(50%)_* ~ 369–377	melt mixing/compression moulding	films	[189]

* Plasticizer/coupling agent/crosslinker/filler. ** ***TS*** = tensile strength; ***E*** = tensile/elastic modulus; ***E_c_*** = complex modulus; ***σ*** = flexural strength; ***E_bend_*** = flexural modulus; ***YS*** = yield strength; ***σ_break_*** = stress at break; ***IS*** = impact strength; ***E_r_*** = reduced elastic modulus (nanoindentation); ***H_i_*** = indentation hardness; ***E_comp_*** = compressive modulus, ***ε_break_***= elongation at break; ***ε_max_*** = maximum stress elongation; ***L_max_*** = maximum load; ***T_n_*** = tenacity; ***S_n_*** = stiffness (nanoindentation). *** ***T_g_*** = glass transition temperature; ***T_g(α)_*** = glass transition temperature obtained at the tan delta maximum using DMA; ***T_m_*** = melt temperature; ***T_c_*** = crystallization temperature; ***T_d_*** = temperature at the onset peak of mass loss; ***T_d(max)_*** = temperature at the peak maximum of mass loss; ***T_d(5%)_*** = temperature at 5 wt.-% mass loss; ***T_d(10%)_*** = temperature at 10 wt.-% mass loss; ***T_d(50%)_*** = temperature at 50 wt.-% mass loss. **** Use or final application of the system, as described by the source reference.

**Table 2:** Summary of thermomechanical properties and preparation/processing conditions of keratin/elastomer and keratin/thermoset polymer systems.

Associated Polymer	Source	Amount (wt.-%)	Additives*	Mechanical Properties (MPa) **	Thermal Properties (°C) ***	Preparation Method	Obs. ****	Ref.
**XNBR**	hair	5	ZnO\EG	*E* = 2200–3000; *TS* ~ 11.4–14.4; *ε_break_* = 371–406(%); *H_s_* = 54–58(N)	*T_g_* = 3.0–6.6	roll-milling/vulcanization	elastomer film	[217]
**SBR**	hair	5–10	ZnO	*TS* ~ 1.4–2.6; *ε_break_* = 134–156(%)	*T_d(5%)_* = 235–250; *T_d(50%)_* = 395–405	roll-milling/vulcanization	elastomer film	[218]
	feather	1–5	TPS/EVOH	*E* = 3000–5000	*T_g_* = (−95)–(−82) and (−4)–131; *T_d(5%)_* ~ 250–300; *T_d(50%)_* ~ 430	melt-mixing/compression moulding	elastomer film	[222]
**NBR**	hair	5–30	ZnO/MMC	*TS* ~ 1.9–5.6; *ε_break_* = 350–895(%)	*T_g_* ~ −50; *T_d(5%)_* = 280–335; *T_d(50%)_* = 410–420	roll-milling/vulcanization	elastomer film	[219]
**XSBL**	hair	-	cotton fabric	film [*TS* ~ 11–13; *ε_break_* = 310–345(%); *H_s_* = 53–55(°)]. coating [*TS* ~ 24–26; *ε_break_* ~17(%); *H_s_* = 52–55(°)	film [*T_g_* ~ −8.5; *T_d(5%)_* ~ 340; *T_d(50%)_* ~ 428]. coating [*T_g_* ~ 85; *T_d(5%)_* ~ 306; *T_d(50%)_* = 333–362]	coating/solvent casting	fibre coating	[220]
**Epoxy (DGEBF)**	feather	11–69 (vol.-%)	diamine/E-glass	*E* ~ 3500–10500; *σ* = 50–310; *E_bend_* = 2350–13400; *ε_break_* = 2.5–6.0(%)	*T_g_* ~ 120–130	moulding	films	[232]
**Epoxy (DGEBA)**	feather	1–7	TETA/CR	*E* ~ 521–1126; *TS* ~ 16–38; *σ_comp_* ~ 55–81; *E_bend_* = 3010–4420; *σ* = 57–79; *IS* = 2.06–3.43(kJ/m^2^)	-	hand lay-up	coating	[234]
**Epoxy (AESO)**	feather	5–45	glass fibre	*E* = 1291–2085; *L_max_* = 97–131(N); *E_f_* = 1.61–1.98(kJ/m^2^); *E_bend_* = 971–1938; *σ* ~ 36–58	*T_g_* ~ 70	moulding	mats	[235]
		5–32	MLAU	*E_s_* = 20–300(at 25°C); *E* = 10–150(at 25°C); *E_f_* = 2.2–7.5(kJ/m^2^)	*T_g_* = 23–29	moulding	films	[240]
**Epoxy (PAESO)**	feather	~30 (vol.-%)	E-glass	*E_bend_* = 8860–10570; *σ* ~ 84–100; *ε_break_* ~ 1.23(%)	*T_g_* ~ 107–112; *T_d(5%)_* = 308–324 306; *T_d(50%)_* = 391–414	compression moulding	circuit board	[236]
**Urea-formaldehyde**	feather	25–75	sawmill hardwood residue	*TS* = 0.65–1.85; *σ* = 2.4–3.2; *σ_comp_* = 0.86–1.42; τ = 0.80–0.98;	-	compression moulding	particle board	[246]
**Phenol-formaldehyde**	feather	2.5–95	wood fibre	*E_bend_* = 1470–3170; *σ* ~ 11–25	-	compression moulding	MDF	[253]
		~33	wood fibreboard	*E_bend_* = 2339–3179; *σ* ~ 32–43; *IB* = 0.29–0.76	-	compression moulding	MDF	[254]

*Plasticizer/coupling agent/crosslinker/filler/scaffold. ** ***TS*** = tensile strength; ***E*** = tensile/elastic modulus; ***E_s_*** = storage modulus; ***E_c_*** = complex modulus; ***σ*** = flexural strength; ***E_bend_*** = flexural modulus; ***YS*** = yield strength; ***σ_break_*** = stress at break; ***IS*** = impact strength; ***E_f_*** = fracture energy; ***E_r_*** = reduced elastic modulus (nanoindentation); ***H_i_*** = indentation hardness; ***H_s_*** = Shore’s hardness; ***E_comp_*** = compressive modulus, ***σ_comp_*** = compressive strength, ***ε_break_***= elongation at break; ***ε_max_*** = maximum stress elongation; ***L_max_*** = maximum load; ***T_n_*** = tenacity; ***S_n_*** = stiffness (nanoindentation); **τ** = shear strength; ***IB*** = internal bonding strength. *** *T_g_ =* glass transition temperature; ***T_d(5%)_*** = temperature at 5 wt.-% mass loss; ***T_d(50%)_*** = temperature at 50 wt.-% mass loss. **** Use or final application of the system, as described by the source reference.

**Table 3:** Summary of thermomechanical properties and preparation/processing conditions of keratin/carbohydrates polymer systems.

Polymer Source	Keratin Source	Keratin (wt.-%)	Additives *	Mechanical Properties (MPa) **	Thermal Properties (°C) ***	Preparation Method	Obs. ****	Ref.
**lignocellulose**	feather	30–35	glycerol	*E* = 36–60; *σ_break_* ~ 1.8–4.3; *ε_break_* = 0.076–0.171(cm/cm)	*T_d(max)_* ~ 300-330;	compression moulding	films	[260]
**CNC**	feather	90–99	-	*E* ~ 451; *σ_break_* ~ 5–23; *ε_break_* ~ 8–28(%)	-	solution casting	films	[261]
			chitosan/glycerol	*TS* ~ 4.6–5.3; *ε_break_* ~ 21.1–27.6(%)	*T_g(α)_* ~ 35–65; *T_m_* ~ 229–254; *T_d(max)_* ~ 310	compression moulding	films	[262]
**wood cellulose**	feather	22–58	glycerol	*E* ~ 149–544; *YS* ~ 11–28; *ε_break_* ~ 45-94(%)	*T_d(max)_* ~ 300–360	solution casting	films	[263]
		10–70	ILs	*E* ~ 6900–7500; *TS* = 88.4–142.4; *ε_break_* ~ 9.0–19.3(%); *S_n_* = 5917–7166(N/m); *T_n_* = 20–46(cN/tex)	-	wet-spinning	Fibres/filaments	[269]
	wool	25–75	ILs	*TS* ~ 9–38	*T_d_* ~ 270–305	solution casting	films	[270]
**cotton cellulose**	wool	5–25	ILs	*E* ~ 1610–1790; *σ_break_* ~ 25–48; *ε_break_* ~ 1.3-3.3(%)	*T_d_* ~ 227–278	co-solvent coagulation	films	[266]
	feather	2.5–20	ILs	*E* ~ 1640–1730; *σ_break_* ~ 26–53; *ε_break_* ~ 4.5–11.6(%)	*T_d_* ~ 278–289	co-solvent coagulation	films	[267]
		5–20	ILs	*E* ~ 5540–17220; *σ_break_* ~ 75–132; *ε_break_* ~ 1.4–7.1(%)	-	wet-spinning	fibres	[268]
**chitosan**	Wool	-	-	*σ_break_* ~ 13–20; *ε_break_* ~ 26–45(%)	*T_m_* ~ 230	Crosslinking	fibre coating	[277]
		-	cellulose	[recovered properties] *σ_break_* ~ 17; *ε_break_* ~ 42(%)	-	solution coating	fibre coating	[278]
		77–90	glycerol	*E* ~ 14–735; *TS* ~ 3–37; *ε_break_* ~ 4–31(%)	-	solution casting	films	[93]
		~10	Pluronic F127/genepin/laponite	*G’* = 2.5–71.3	-	Crosslinking	Injectable hydrogel	[283]
		5–15	PCL/PEO	*TS* ~ 3.2–5.3; *ε_break_* ~ 10–63(%); *E_break_* = 0.34–2.34(J/m^3^)	-	Electrospinning	fibres	[284]
	Feather	5–20	sorbitol/starch	*E* ~ 241–1142	*T_g(α)_* ~ 159–196; *T_m_* ~ 200; *T_d(50%)_* ~ 300–340	solution casting	films	[275]
		5–10	sorbitol/starch	*E_s_* ~ 1158–2972; *E* ~ 29–826; *σ_max_* ~ 2.0–15.6	*T_g(α)_* ~ 70–104; *T_d(50%)_* ~ 290–300	solution casting/melt-mixing	films	[276]
		1–15	-	*TS* ~ 40–65; *ε_break_* ~ 10–15(%)	*T_d(max)_* ~ 277–331	solution casting/coagulation	membranes	[282]
	Hair	25–50	-	*TS* ~ 22–28	-	Crosslinking	membranes	[285]
	Hoof	25	gelatine	*E_comp_* = 0.005–0.009; *E* ~ 0.010–0.096	*T_d(50%)_* ~ 361	freeze-drying	scaffold	[286]
	Horn	67	-	*TS* ~ 1.6; *ε_break_* ~ 21.6(%); *L_max_* = 6.3(N); *ε_max_* = 5.12(mm)	*T_m_* ~ 216; *T_c_* ~ 180	freeze-drying	scaffold	[287]
		25–50	ethylene glycol	*E* ~ 0.5–3.2; *TS* ~ 7.4–21.1; *ε_break_* ~ 6.2–16.0(%)	*T_d_* ~ 300; *T_m_* ~ 145–175	solution casting	films	[288]
**alginate**	Feather	10	glycerol	*E* ~ 0.08–0.38; *ε_break_* ~ 31–60(%)	-	solution casting	films	[292]
		50–90	sorbitol	*E* ~ 6.0–16.3; *ε_break_* ~ 25–29(%)	*T_c_* = 211–218	solution casting	films	[293]
**starch**	Hair	15–30	APP	*E* ~ 165–247; *TS* ~ 10–13; *T_n_* = 2–19; *ε_break_* ~ 26–190(%); *IS* = 81–397(J/m)	*T_d(5%)_* ~ 211–250; *T_d(max)_* ~ 361–397;	melt-mixing	flame retardant	[297]

*Plasticizer/coupling agent/crosslinker/filler/scaffold. ** *TS* = tensile strength; *E* = tensile/elastic modulus; *G’* = shear storage modulus; ***YS*** = yield strength; ***σ_break_*** = stress at break; ***σ_max_*** = maximum stress; ***IS*** = impact strength; ***E_comp_*** = compressive modulus, ***ε_break_***= elongation at break; ***ε_max_*** = maximum stress elongation; ***L_max_*** = maximum load; ***T_n_*** = tenacity; ***E_break_*** = energy at break (toughness); ***S_n_*** = Stiffness (nanoindentation). ***T_g_ *=* glass transition temperature; *T_g(α)_*= glass transition temperature obtained at the tan delta maximum using DMA; ***T_m_*** = melt temperature; ***T_c_*** = crystallization temperature; ***T_d_*** = temperature at the onset peak of mass loss; ***T_d(max)_*** = temperature at the peak maximum of mass loss; ***T_d(50%)_*** = temperature at 50 wt. % mass loss.

**Table 4:** Summary of thermomechanical properties and preparation/processing conditions of keratin/protein polymer systems.

Polymer Source	Keratin Source	Keratin (wt.-%)	Additives *	Mechanical Properties (MPa) **	Thermal Properties (°C) ***	Preparation Method	Obs. ****	Ref.
**gelatine**	wool	-	TGas	*TS* ~ 26.8 (270–360N)	-	crosslinking	fibre coating	[300]
	hair	33–67	-	-	*T_m_* ~ 250; *T_d(max)_* ~ 310–340;	solution casting	films	[301,302]
	n.d.	10	glutaraldehyde	*E* ~ 0.23	-	freeze-drying	scaffolds	[303]
**soy gluten**	feather	3–9	glycerol	*E* ~ 97–109; *TS ~* 4.8–9.5; *ε_break_* = 94–110(%)	*T_g(α)_*~ −30/55; *T_d(max)_* ~ 310	solution casting/compression moulding	films	[311]
**Silk fibroin**	wool	20–50	-	*E* = 1409–2724; *TS* = 9153–29755; *ε_break_* = 0.36–0.68(mm)	*T_g_*~ 178; *T_m_*~ 224–278; *T_c_* ~ 226; *T_d_* ~ 274–296	solution casting	films	[94]
		10–90	-	-	*T_g_*~ 154–171; *T_d_* ~ 274–300;	crosslinking	hydrogels	[314]
		0.7–2.36	PVF/cotton	*T_n_* ~ 2.1–2.6(cN/dtex); *ε_break_* ~ 23.4–30.2(%)	-	crosslinking	fibre	[315]

*n.d.* = not described; *Plasticizer/coupling agent/crosslinker/filler/scaffold. ** ***TS*** = tensile strength; ***E*** = tensile/elastic modulus; ***ε_break_*** = elongation at break; ***T_n_*** = tenacity. *** ***T_g_*** *=* glass transition temperature; ***T_g(α)_*** = glass transition temperature obtained at the tan delta maximum using DMA; ***T_m_*** = melt temperature; ***T_c_*** = crystallization temperature; ***T_d_*** = temperature at the onset peak of mass loss (TGA) or decomposition endotherm (DSC); ***T_d(max)_*** = temperature at the peak maximum of mass loss.
